# Neuronal activity-driven 3D chromatin dynamics in cortical pyramidal neurons depend on SATB2

**DOI:** 10.1093/nar/gkag788

**Published:** 2026-08-25

**Authors:** Nico Wahl, Mujahid Ali, Sergio Espeso-Gil, Georg Dechant, Galina Apostolova

**Affiliations:** Institute for Neuroscience, Medical University of Innsbruck, Innsbruck 6020, Austria; Institute for Neuroscience, Medical University of Innsbruck, Innsbruck 6020, Austria; Institute for Neuroscience, Medical University of Innsbruck, Innsbruck 6020, Austria; Institute for Neuroscience, Medical University of Innsbruck, Innsbruck 6020, Austria; Institute for Neuroscience, Medical University of Innsbruck, Innsbruck 6020, Austria

## Abstract

Neuronal activity induces widespread changes in chromatin organization, yet the mechanisms of activity-dependent 3D genome remodelling remain incompletely understood. Here, we identify SATB2 as a key regulator of activity-dependent 3D chromatin dynamics in cortical pyramidal neurons. Using primary cultures from floxed and *Satb2* conditional knockout mice, we combined Hi-C and chromatin accessibility mapping to capture rapid 3D epigenome reorganization following neuronal stimulation. Within 1 h of activation, floxed neurons exhibited enhanced chromatin interactions, increased chromatin accessibility, and the emergence of a novel activity-dependent (AD) compartment enriched for SATB2 binding sites. The AD compartment harbours metabolic and housekeeping genes that undergo transient repression upon neuronal activation, thereby prioritizing translation of long synaptic transcripts. Loss of SATB2 disrupted these activity-induced changes, including chromatin accessibility, long-range interactions, and AD compartment formation, resulting in attenuated induction and repression of key activity-regulated genes. These findings establish SATB2 as a central organizer of activity-dependent chromatin architecture dynamics, required for coordinated activation and repression of activity-regulated genes in cortical pyramidal neurons.

## Graphical Abstract

**Graphical Abstract:**
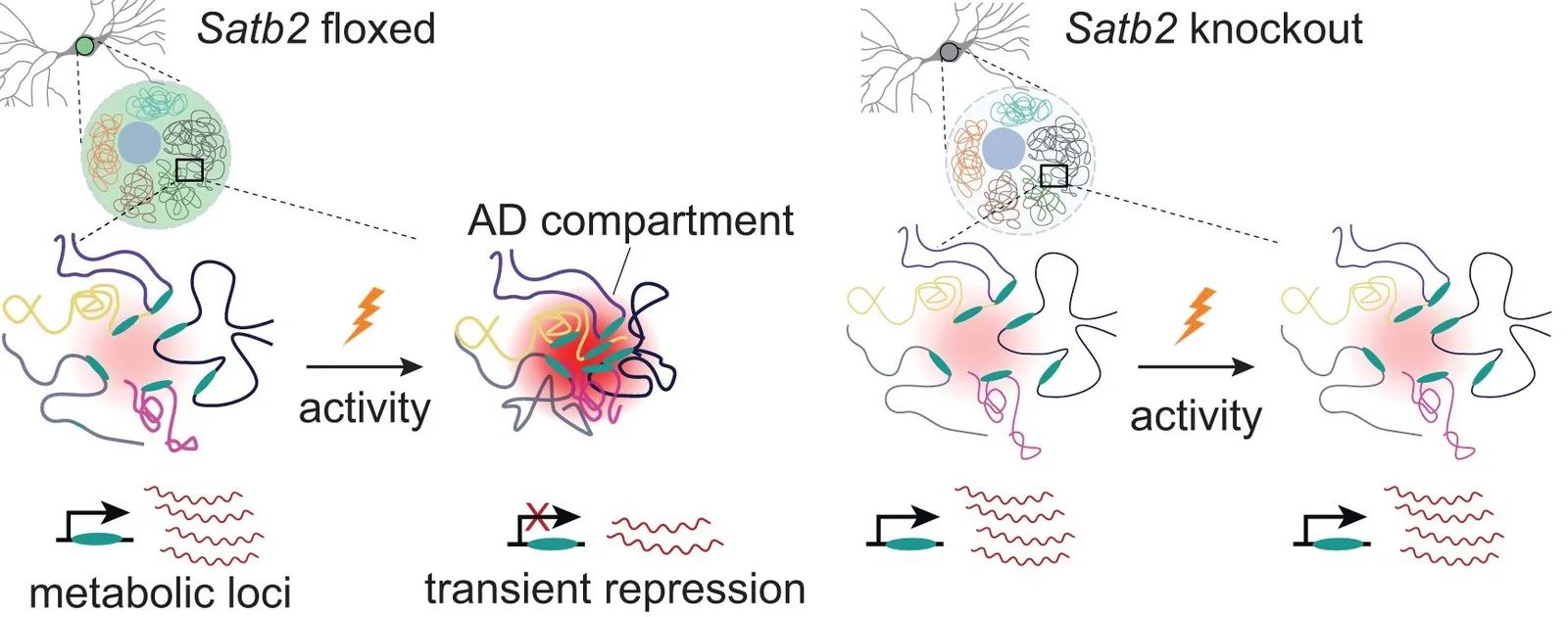

## Introduction

Neuronal activity profoundly reshapes gene regulatory networks in the brain, driving adaptive processes such as synaptic plasticity, learning, and memory [1]. Stimulation triggers waves of activity-regulated genes (ARGs), including immediate early genes (IEGs), primarily transcription factors (TFs) such as *Fos, Egr1*, and *Npas4*, followed by late response genes (LRGs), which encode effector proteins involved in synaptic remodelling [2]. In parallel, a distinct set of genes, functionally far less explored than IEGs and LRGs, undergoes transient downregulation [3].

Previous studies have highlighted a complex interplay between multiple chromatin-based regulatory mechanisms in mediating the induction of ARGs [4]. One such mechanism is chromatin looping, which brings enhancers and promoters of ARGs into close proximity. IEGs typically engage with enhancers via short-range, rapidly formed loops that precede peak messenger RNA (mRNA) expression, whereas LRGs rely on slower, long-range looping events that emerge hours after stimulation [5]. Beyond DNA looping, additional chromatin-dependent epigenetic processes have recently been implicated in ARG expression, including chromatin remodelling and three-dimensional (3D) genome reorganization [6–10]. Large-scale compartment switching—from inactive to active chromatin, together with changes in promoter–enhancer interactions, has been associated with memory consolidation in the adult hippocampus *in vivo* [11].

However, the dynamics and scope of 3D epigenome remodelling following neuronal stimulation remain poorly understood. Key questions include how rapidly these structural changes occur and how higher-order genome architecture, beyond compartment switching and chromatin looping, such as long-range TAD–TAD interactions or inter-chromosomal contacts, is affected. In addition, most studies to date have focused on chromatin regulatory mechanisms underlying IEG and LRG induction [12], whereas the 3D epigenomic mechanisms governing gene repression, and how these are temporally coordinated, remain largely unexplored.

Another critical but unresolved question concerns the chromatin scaffolding proteins that mediate activity-driven 3D genome alterations. While ubiquitous organizers such as CTCF and cohesin contribute broadly to chromatin architecture in all cell types, including neurons [13], neuronal cells may also rely on specialized proteins to regulate chromatin topology in a subtype-specific or stimulus-specific manner. We have previously demonstrated a pivotal role for SATB2, a nuclear matrix-associated protein selectively expressed in pyramidal neurons of cortex and hippocampus [14–16], in orchestrating 3D genome folding and chromatin accessibility in this neuronal subtype [17]. SATB2 interacts with ubiquitous 3D genome organizers such as CTCF and regulates the 3D chromatin structure of roughly 50% of cognition-related loci, including ARGs, in a highly specific manner, acting across multiple levels of 3D chromatin architecture [17]. Consistent with this, mice lacking SATB2 in the adult nervous system exhibit cognitive impairments, which are rescued by viral re-expression of SATB2 in conditional knockout (cKO) mice [18].

What remains unclear is whether SATB2 also contributes to activity-driven 3D genome remodelling and stimulus-specific transcriptional responses. In the present study, we address this question by examining the role of SATB2 in 3D chromatin dynamics triggered by moderate, near-physiological neuronal stimulation, using primary cortical neurons derived from floxed and *Satb2* cKO mice. This model offers several advantages, including a relatively homogeneous culture of up to 90% pyramidal neurons, uniform SATB2 expression in all floxed pyramidal cells, and precise temporal control over neuronal activity in all neurons.

Using this approach, we identify a novel activity-induced chromatin compartment in floxed neurons, which we term the “AD compartment,” that supports coordinated, temporal down-regulation of metabolic genes upon neuronal stimulation. By comparing transcriptomic and 3D epigenomic profiles of control and *Satb2* cKO cortical neurons, we demonstrate a critical role for SATB2 in activity-induced chromatin compaction, increased chromatin accessibility, and AD compartment formation. These findings provide a mechanistic link between SATB2-mediated 3D chromatin modifications and activity-driven transcriptional homeostasis in cortical neurons.

## Materials and methods

### Mouse models

All experimental procedures were approved by the Austrian Animal Experimentation Ethics Board. The following mouse lines were used: Satb2^flx/flx^ [18] and Satb2^flx/wt^::Nes-Cre [19]. Mice were housed under a 12 h light/12 h dark cycle at 22.5°C and 55 ± 10% humidity, with *ad libitum* access to food and water.

### Primary cortical cultures

Cortices were dissected from neonatal Satb2^flx/flx^::Nes-Cre and Satb2^flx/flx^ mice of mixed sex and after dissociation for 10 min with papain (Worthington Biochemical Corporation, 34U/ml) cortical neurons were plated at a density of 0.125 × 10^6^ cells/cm^2^ in 60-mm culture dishes, 6-well plates, or 12-well plates. All culture surfaces were pre-coated with poly-L-ornithine (Sigma; 0.5 mg/ml, overnight at 4°C) followed by laminin (Sigma; 0.005 mg/ml, 2 h at 37°C).

Cells were initially cultured in minimal essential medium (Gibco) supplemented with 10% horse serum (Gibco), 1% penicillin/streptomycin (Thermo Fisher Scientific), and sodium pyruvate (Sigma–Aldrich). After 2 h of pre-incubation, the medium was replaced with Neurobasal A (NBM-A, Gibco) supplemented with GlutaMAX, penicillin/streptomycin, and B27 (Thermo Fisher Scientific). Neurons were maintained for 14 days at 37°C in a humidified incubator with 5% CO_2_. To inhibit glial proliferation, cytosine arabinoside (Ara-C, 5 µM, Sigma) was added at day *in vitro* 3 (DIV3). On DIV4, half of the medium was replaced. At DIV13, neurons were silenced by treatment with NBQX (20 µM, Tocris) for 16 h. For activation studies, neurons were pre-treated with NBQX as described and subsequently stimulated on DIV 14 with bicuculline (Bic, 50 µM, Tocris) for either 1 h or 6 h.

### Viral transduction

For acute *Satb2* depletion, primary cortical cultures derived from Satb2^flx/flx^ mice were transduced at DIV6 with ready-to-use AAV8 particles produced from pENN.AAV.hSyn.HI.eGFP-Cre.WPRE.SV40 (Addgene, #105540) or pAAV-hSyn-EGFP (Addgene, #50465) as a control. For rescue experiments, primary cortical neurons derived from Satb2^flx/flx^::Nes-Cre mice were transduced on DIV4 with serotype AAV8 viral particle produced from pAAV-hSyn-V5-mSatb2 [17] and pAAV-hSyn-EGFP (Addgene, #50465) as a control. For all viral transductions a titer of 3.22 × 10^4^ VG/cell has been used.

### Nuclei isolation

On DIV14, primary cortical neurons were washed twice with room temperature 1× phosphate-buffered saline (PBS). Nuclei were isolated by adding NEB [20 mM HEPES-KOH, pH 7.9, 10 mM KCl, 0.5 mM spermidine, 0.1% Triton X-100, 20% glycerol, and one cOmplete ethylenediaminetetraacetic acid (EDTA)-free protease inhibitor tablet (Roche)]. Cells were incubated on a shaker at 4°C for 7 min, then detached using a cell lifter and gently resuspended with a P1000 pipette. The cell suspension was transferred to a fresh Eppendorf tube and centrifuged at 600 × *g* for 3 min at 4°C. After carefully removing the supernatant, the pellet was resuspended in Wash Buffer (Active Motif). Intact nuclei were stained with Trypan Blue (Thermo Fisher Scientific) and counted using a Neubauer chamber. For each CUT&Tag reaction, 150 000 nuclei were used.

### Immunoblotting

Cultured cortical neurons were harvested at DIV14. Cells were washed twice with 1 ml 1× PBS and lysed in 2× Roti-Load buffer (Roth, Cat. No. K929.1) using a cell scraper. The lysates were sonicated (Branson Ultrasonics™ Sonifier 250 CE; output control 6, duty cycle 30%, two pulses of 2 s each) and centrifuged for 5 min at 13 000 rcf and 4°C. Samples were subsequently boiled for 5 min at 95°C and briefly centrifuged. Prepared samples, together with a protein ladder (Precision Plus Protein Dual Color; Bio-Rad, Cat. No. 1610374), were loaded onto a Novex™ Tris-Glycine Mini Protein Gels, 12%, 1.0 mm, WedgeWell™ format (Biorad, Cat. No. XP00120) and run at 80 V for ~3 h. Proteins were transferred to a PVDF membrane (Millipore, Cat. No. IPVH00010) using semi-dry electroblotting for 30 min at 25 V. Successful transfer was confirmed by staining the membrane with Ponceau S solution (Sigma–Aldrich, Cat. No. P7170) for 5 min at room temperature. Membranes were rinsed with TBS-T [1 ml Tween 20 (Roth, Cat. No. 9127) in 1 l TBS] and cut according to the molecular weights of the target proteins. Membrane sections were blocked in blocking buffer [5% milk powder (Roth, Cat. No. T145.4) in TBS-T] for 1 h and incubated with primary antibodies (c-Fos, Cell Signaling, Cat. No. 2250S, SATB2, Abcam, Cat.No. ab92446, NPAS4, [20]) diluted in blocking buffer overnight at 4°C. The following day, membranes were washed three times with washing buffer (20 ml blocking buffer in 80 ml TBS-T, 1:5) and incubated with corresponding HRP-conjugated secondary antibodies (Cell Signaling, Cat. Nos. 7074 and 7076) diluted in washing buffer for 1 h at room temperature. After three additional washes in washing buffer, membranes were briefly rinsed with TBS-T and developed using ECL reagent (Bio-Rad, Cat. No. 1705062). Chemiluminescent signals were detected using the ChemiDoc imaging system (Bio-Rad), and protein expression was quantified with Image Lab software version 6.1.0 build 7 (Bio-Rad).

### Hi-C

*In situ* Hi-C was performed using the Arima-HiC kit, following the manufacturer’s protocol. In brief, 2.5 × 10⁶ primary cortical neurons were fixed directly in culture dishes by adding formaldehyde to a final concentration of 1% for 10 min at room temperature. Cells were then washed twice with 1× PBS, detached using a cell lifter, and collected in 1× PBS supplemented with protease inhibitors. The cell suspension was centrifuged at 500 × *g* for 10 min at 4°C, and the resulting pellets were snap-frozen in liquid nitrogen.

Following nuclei isolation and chromatin digestion, biotin was incorporated and DNA ends were ligated. After reverse crosslinking and purification, the DNA was sheared to an average fragment size of 400 bp using a Covaris M220 sonicator (duty factor: 12%, peak incident power: 75, cycles per burst: 200, duration: 60 s). Size selection was performed using AMPure XP beads as recommended in the Arima-HiC kit manual.

Hi-C libraries were prepared using the Swift Biosciences Accel-NGS 2S Plus DNA Library Kit in accordance with the Arima-HiC User Guide (November 2018 version). Libraries were sequenced on two platforms: Illumina HiSeq 2500 (125 bp paired-end, 250 million reads per sample, *n* = 2) at the New York Genome Center, and Illumina NovaSeq (150 bp paired-end, 650 million reads per sample, *n* = 4) at Novogene UK.

### Hi-C data analysis

#### Mapping, filtering, normalization, and quality control

Sequencing reads were mapped to the mouse reference genome assembly (mm10), and the resulting alignments were filtered for artifacts and ICE-normalized using default parameters in HiC-Pro (v.2.11.4) [21]. For some analyses requiring specific balancing, such as the differential mapping below, KR (Knight-Ruiz) normalization was used [22].

Data reproducibility between replicates was assessed using HiCRep method [23] with a wrapper function (HiCReproducibility.R, available at https://github.com/sespesogil/SATB2_3D_Genome/blob/main/HiCReproducibility). Briefly, Hi-C matrices were generated at 250 kb resolution, and pairwise reproducibility values (SCC scores) were computed using the *get.scc* function with parameters: h = 1, lbr = 0, ubr = 5 000 000. The resulting SCC matrix was then visualized using the pheatmap R package [24].

#### Hi-C matrix visualization

General visualization of Hi-C matrices was performed using Juicebox (https://github.com/theaidenlab/juicebox). To visualize specific chromatin interaction changes between Bic and NBQX treatments, differential contact matrices were generated for representative genomic regions using the GENOVA R package (v1.0.0). Balanced Hi-C contact matrices at 100 kb resolution were loaded from .cool files using the load.contact function. A differential interaction map was then generated using the hic.matrixplot function with the “diff” mode option. This procedure subtracts the NBQX contact matrix from the Bic matrix on a bin-by-bin basis. To enhance visualization and prevent extreme values from dominating the colour scale, the differential matrix values were thresholded at ±25.

#### Decay plots and quantification of intra- and inter-chromosomal interactions

We calculated the expected contact probability using the FAN-C tool subcommand “*fanc expected*” [25]. This analysis generates an expected contact matrix in which each genomic bin represents the average number of contacts between two loci at a given genomic distance, averaged across the entire genome, and visualizes these values as contact probability [P(s)] decay curves. For this analysis, we used ICE-normalized Hi-C data from all samples at 100 kb resolution. The expected contact probabilities were computed using the following command: fanc expected -l s1 s2 s3 s4 -p 100Kb_decayplot.pdf --mpl "plt.xlim(1000, 2000000); plt.ylim(1000, 100000)" input s1 s2 s3 s4 output contacts.txt. Genomic distance bins ranged from 100 kb to 20 Mb.

We used HiC-Explorer function hicAggregateContacts on “intra-chr” or “inter-chr” mode to obtain the corresponding interaction frequency at 100 kb resolution.

#### A/B compartment analysis

To visualize genome compartmentalization, Saddle plots were generated using “*saddle*” function followed by “*visualize*” from the GENOVA R package [26] (https://github.com/robinweide/GENOVA). To distinguish A versus B compartments, we used P0 forebrain H3K27ac active mark from ENCODE (ENCSR094TTT).

Differential A/B compartment analysis was carried out by using *dcHiC* [27] (v2.1). Input files were prepared following dcHiC guidelines to obtain observed versus expected (O/E) correlation matrices (https://github.com/ay-lab/dcHiC/tree/master). Briefly, Hi-C files at 100-kb-resolution were first preprocessed using “preprocess.py” running in python v3.7.10. Output was used as input for “*dchic.py*” script to compute compartment differences between samples.

To annotate genes residing in differential compartments, we used *annotatr* package [28].

#### AD compartment analysis

To identify the activity-dependent (AD) chromatin compartment, Hi-C interaction matrices were processed through a multi-step pipeline integrating data format conversion, normalization, matrix comparison, and principal component analysis (PCA). Initially, Hi-C data stored in .hic format were converted to .cool format using the Juicer tool. The resulting .cool files were subsequently transformed into .h5 format using the hicConvertFormat function from the HiCExplorer suite to facilitate downstream analyses. Each Hi-C contact matrix was normalized to an observed-over-expected (O/E) format using the hicTransform function, enabling the removal of distance-dependent contact decay and allowing for the comparison of relative interaction frequencies. Differential interaction maps were generated using the browserhicCompareMatrices function of HiCExplorer [29], applying the log_2_ ratio operation log_2_ (Bic/NBQX). This operation was performed separately for both genotypes, at 100 kb resolution.

The resulting differential matrices were converted back into .cool format. To ensure matrix balancing prior to PCA, we calculated balancing weights using cooler balance, as implemented in the compD Snakemake pipeline (https://github.com/LegubeDNAREPAIR/compD_pipeline_python). Next, PCA was performed on the balanced, differential O/E matrices using a custom Python script (process_pca_compD.py). The first Eigenvector (EV1) was extracted for each genotype. Regions with positive EV1 values greater than 0.05 (EV1 > 0.05) were operationally defined as AD compartments, representing chromatin domains with the strongest increase in the interaction frequency in response to neuronal stimulation.

For visualization, AD compartments were exported as signal tracks in bigWig format using the UCSC bigWig conversion utilities, enabling integration with *pyGenome* tracks tools [30].

To validate that the identified AD-compartment signal represents a true biological change and is not an artifact of technical variability, we performed a “within-condition” control analysis. The six biological replicates for each condition (Bic and NBQX) were first partitioned into two independent, depth-matched pools (*n* = 3 replicates per pool). We then applied our standard AD-compartment calling pipeline to compare these replicate pools against each other: Bic pool 1 versus Bic pool 2; NBQX pool 1 versus NBQX pool 2. Critically, this control analysis used parameters identical to those in the primary experimental comparison (Bic versus NBQX). Specifically, Hi-C matrices were binned at 100 kb, normalized, and processed with the same eigenvector-based scoring method. For visualization, the scores from both the primary analysis (Bic versus NBQX) and the within-condition controls were plotted for each 100 kb genomic bin according to its chromosome position using ggplot2 in R.

#### Topologically associating domains and TAD cliques

Topologically associating domains (TADs) were identified at a 25 kb resolution using SpectralTAD [31]. In short, ICED-normalized matrices generated by HiC-Pro were converted to “bedpe” format using the “hicpro2bedpe” function from HiCcompare [32]. The pooled sample data were then input into SpectralTAD, which was executed iteratively for each chromosome. To compute the interaction density within TADs and their 1,2,… , N neighbours, “TAD + *n*” analysis was used. Briefly, “TAD + *n*” plots were generated using “*intra_inter_TAD*” followed by “*visualize*” functions of GENOVA R package. Intra-TAD interactions were quantified using “*hicInterIntraTAD*” function in HiCExplorer.

TAD cliques were identified using a custom Python script built upon the NetworkX library (https://networkx.org/) to model chromatin interactions as undirected graphs. In this representation, each node corresponds to a TAD, and edges denote statistically significant pairwise TAD–TAD interactions derived from Hi-C contact matrices. Clique structures—defined as fully interconnected groups of TADs—were detected using activity-based clustering. The maximum clique size was set to *k* = 7, and each TAD was assigned to its largest associated clique. Graphs were visualized using the *igraph* package and plotted using *pheatmap* R package.

#### Insulation score analysis

Insulation score analysis was performed using the *fanc insulation* command from the FAN-C suite [25] on Hi-C contact matrices binned at 100 kb resolution. Consistent with the upstream compartment analysis, matrices were normalized using the default chromosome-average method and subsequently log-transformed. Unless otherwise stated, all parameters were kept at their default settings.

To generate aggregate metaprofiles of insulation at compartment boundaries, the calculated insulation scores were exported to BigWig format. Reference regions for the analysis were defined as 30 kb windows centered on each boundary of the identified AD-compartment bins (±15 kb). Using the deepTools suite, the *computeMatrix* command summarized insulation scores from Bic and NBQX conditions across a ±1-Mb window centered on each boundary. As a negative control, an identical analysis was performed on an equal number of randomly selected genomic locations. Finally, average insulation profiles were generated and visualized using *plotProfile*.

#### Sub-compartment identification

Sub-compartments were defined by k-means clustering of epigenetic features within 10 kb non-overlapping genomic windows (chrM, X, and Y were excluded). We generated enrichment profiles for H3K4me3 (GSM5738212), H3K27me3 (this study), H3K9me3 (GSM5738214), Input (GSM5738208), and Lamin B1 [33]. These genomic windows were first segregated by their primary A/B compartment status, and k-means clustering (with an initial *k* = 8) was performed independently on the A and B compartment groups. After removing noisy outlier clusters, the remaining clusters were manually curated and merged to define five stable sub-compartments: A1, A2, B1, B2, and B3. Annotation was based on canonical marker enrichment (e.g. A1-H3K4me3, B1-H3K27me3, B3-Lamin B1) and further supported by gene density and expression data (not shown). Finally, the AD compartments identified in this study were intersected with these five sub-compartment classes to determine their epigenetic composition.

#### Chromatin loops

Chromatin loops were called by Mustache (v1.2.0), a local enrichment-based loop calling method [34]. Replicate Hi-C matrices were pooled to be able to perform the analysis at 5 kb resolution. The following parameters for differential loop detection were used: *P*-value (-p) of .05 and sparsity (-st) of 0.8, together with KR normalization (“-norm KR” parameter). To assign differential chromatin loops to genes, we intersected loop anchors with gene promoters (defined as sequences <1 kb upstream of the transcription start site (TSS) and 1–5 kb upstream of the TSS) using *annotatr* package [28]. Genes that were assigned to both gained and lost loops were excluded from Gene ontology (GO) enrichment analyses.

#### Hi-C aggregate analysis

To assess average chromatin contact enrichment, we performed loop aggregation analysis using FAN-C (*v0.928*) [25] with *Python 3.12.10*. Aggregation was computed on pairwise genomic regions provided in BEDPE format, using Hi-C contact matrices at 10 kb resolution in .cool format, normalized by the *Knight-Ruiz* (*KR*) method. Loop interaction frequencies were quantified as O/E values.

For aggregation peak analysis, we used the *hicAggregateContact* function of the *HicExplorer* tool (*v3.7.6*), using parameters --operationType as *mean* and --transformation as O/E. The interaction range was selected above the average TAD size (300 kb to 15 Mb). To visualize the resulting aggregated Hi-C matrices, we used the *hicPlotMatrix* function to generate heatmaps on the O/E scale to compare between the samples. For quantification of interaction enrichment, we used the *ggplot2* package in R to produce boxplots.

### ATAC-seq

#### Libraries preparation

For each experiment, 1 × 10^6^ primary cortical neurons were plated in a single well of a six-well plate. Prior to nuclei isolation, cells were washed twice with 1× PBS, followed by the addition of nuclei isolation buffer [1× PBS, 0.1% Triton X-100, 5 mM AEBSF Pefabloc (Sigma), 5 mM sodium butyrate, and one cOmplete EDTA-free protease inhibitor tablet (Roche)]. The plate was incubated on a shaker at 4°C for 7 min, after which the cells were detached using a cell lifter and gently resuspended with a P1000 pipette. The cell suspension was transferred to an Eppendorf tube and centrifuged at 500 × *g* for 10 min at 4°C. The supernatant was discarded, and the pellet was gently resuspended in 1× PBS containing protease inhibitors. Intact nuclei were counted using a Neubauer chamber.

A total of 25 000 nuclei were used for tagmentation in 4× TD buffer (132 mM Tris–acetate, pH 7.8, 264 mM potassium acetate, 40 mM magnesium acetate, 64% dimethylformamide, 0.005% digitonin) along with 1.5 μl of Tn5 transposase (Illumina). The reaction was incubated at 37°C for 30 min and purified using the MinElute Kit (QIAGEN) according to the manufacturer’s instructions. Libraries were prepared as previously described [35], with each library receiving a unique barcode and undergoing 11 cycles of PCR amplification. Post-amplification, libraries were purified using 1× AMPure XP beads (Beckman) and eluted in 33 μl of 10 mM Tris–HCl (pH 8.0). Library concentration was measured using Qubit (Thermo Fisher Scientific), and fragment size distribution was assessed using high-sensitivity D5000 ScreenTapes on a TapeStation (Agilent). ATAC-seq libraries were sequenced by Novogene UK on a NovaSeq platform using 150 bp paired-end reads, achieving a sequencing depth of 150 million reads per replicate (*n* = 3).

#### ATAC-seq analysis

Quality control of raw reads was performed using *FastQC* tool and quality trimming of sequencing reads was performed with *fastp* (v0.25) [36] using the following command: *fastp -i $R1 -I $R2 -o $out.fq1.trimmed.gz -O $out.fq2.trimmed.gz --phred64 --detect_adapter_for_pe --dedup --dup_calc_accuracy 3 --overrepresentation_analysis -R fastp_QC_report*. Samples were subsequently aligned to the *mm10* genome using the *Bowtie2* alignment software bowtie2 (v2.2.9) [37]. The following parameters were used: --very-sensitive setting with parameters (using the rep -e "^@" -e "XM:i:[012][^0–9]" | grep -v "XS:i:" tags created by bowtie2 mapping in the SAM file, we utilized SAMtools to filter out multi-mapped reads, allowing us to retain only uniquely mapped reads) set to permit only unique alignments. Duplicates were subsequently removed using *SAMtools* /1.3.1 *samtools* view *rmdup* [38]. The reproducibility of replicate ATAC-seq libraries was assessed by calculating Pearson correlation between BAM files by using *deepTools2* [39]. Peaks were called from the merged BAM files (*n* = 6) using *MACS3* (v3.0.3) [40] with the following parameters: macs3 callpeak -t $chip -f BAMPE -g mm10 -n $out -p 1e-12 --keep-dup all --slocal 1000 --shift 100 --nolambda. Peaks overlapping blacklisted genomic regions were excluded from downstream analysis. Signal coverage files were generated using the bamCompare function from *deepTools* (v3.5.6) with a bin size of 50 bp (--bs 50 --minMappingQuality 10 --normalizeUsing RPKM). The bedGraph files were converted to bigWig format using bedGraphToBigWig (/ucsc/482/bedGraphToBigWig).

Differential open chromatin regions (OCRs) were identified using *MACS3* with default parameters. First, we merged bam files from three replicates (Bic-treated or NBQX-treated) and we used the merged bam files as input files for MACS3. To detect gained OCRs, Bic-treated merged sample was considered as treatment and NBQX as control (Bic/NBQX). Conversely, for identifying lost OCRs, NBQX-treated merged sample was considered as treatment and Bic-treated as control (NBQX/Bic). Finally, the resulting gained and lost OCRs were subtracted from the merged peak set (*n* = 6) to define invariant OCRs. Merged signal coverage was generated using *deepTools* (*v3.5.6*). For coverage visualization, replicates were merged and processed using bamCoverage with the following parameters: --binSize 50 --normalizeUsing *RPGC* --effectiveGenomeSize *mm10*. For gained ATAC-seq signal coverage, log_2_-fold change coverage tracks were generated using bamCompare (Bic/NBQX) with the parameters: --binSize 50 --minMappingQuality 10 --normalizeUsing *RPKM*. These signal tracks were used for downstream analyses and visualization.

*Homer* (v4.11) was used for TF motif analysis [41]. Peak annotation was done using ChIPseeker [42]. To determine the overlap between differential OCRs and between gained OCRs and SATB2 peaks, OCR summits were extended ±250 bp. Average profiles were generated using deeptools function *computeMatrix*, 50 bp bin size.

### CUT&Tag

The CUT&Tag protocol was performed using the CUT&Tag-IT^®^ Assay Kit (Active Motif, #53160), following the manufacturer’s instructions. For each reaction, 20 μl of Concanavalin A-conjugated beads were activated and added to the freshly extracted nuclei. The suspension was incubated at room temperature (RT) for 10 min. Antibodies were then added to the bead-bound nuclei in Antibody Binding Buffer. Primary antibodies—SATB2 (ab92446, Abcam), H3K27me3 (39 055, Active Motif), and H3K27ac (ab177178, Abcam)—were used at a 1:50 dilution. Beads were incubated with rotation at 4°C for 2 h. Bead-bound nuclei were washed twice with 1 ml cold Dig-Wash Buffer (Active Motif) and resuspended in 100 μl cold Dig-Wash Buffer with the addition of a 1:100 dilution of Guinea Pig Anti-Rabbit secondary antibody (Active Motif). Secondary antibody incubation was carried out for 1 h at RT on a rotator. After incubation, bead-bound nuclei were washed three times with 1 ml cold Dig-Wash Buffer. For pA-Tn5 binding, bead-bound nuclei were resuspended in 100 μl Dig-300 Buffer (Active Motif) with a 1:100 dilution of CUT&Tag-IT™ Assembled pA-Tn5 Transposomes (Active Motif). The suspension was incubated for 1 h at RT on a rotator. After incubation, the nuclei were washed three times with 1 ml Dig-300 Buffer. For the tagmentation reaction, bead-bound nuclei were resuspended in 125 μl Tagmentation Buffer (Active Motif) and incubated at 37°C for 60 min. To stop the tagmentation, 4.2 μl of 0.5 M EDTA, 1.25 μl of 10% SDS, and 1.1 μl of Proteinase K (10 mg/ml) were added, followed by incubation at 55°C for an additional 1 h. DNA was purified using the column purification protocol supplied with the kit.

Libraries were prepared according to the CUT&Tag-IT^®^ Assay Kit (Active Motif, #53160) instructions. Indexing primers provided in the kit were used, and libraries were PCR-amplified for 14 cycles. PCR products were cleaned using 1.1× SPRI beads and eluted in 20 μl DNA Purification Buffer (Active Motif). All libraries were sequenced at Novogene on an Illumina NovaSeq platform (150 bp paired-end sequencing). Each replicate of SATB2 (*n* = 10), H3K27ac (*n* = 2), and H3K27me3 (*n* = 2) CUT&Tag libraries was sequenced to a depth of 10 million reads.

#### CUT&Tag analysis

Raw read quality was assessed using *FastQC*, and adapter trimming and quality filtering were performed using *fastp* (v0.25) with the following parameters: fastp -i $R1 -I $R2 -o $out.fq1.trimmed.gz -O $out.fq2.trimmed.gz --phred64 --detect_adapter_for_pe --dedup --dup_calc_accuracy 3 --overrepresentation_analysis -R fastp_QC_report. To ensure high-quality alignments, non-uniquely mapped reads were subsequently filtered out using SAMtools, and duplicate reads were removed using samtools rmdup (v1.3.1). Peaks were called from merged BAM files (*n* = 10) using *MACS3* (v3.0.3) with the following parameters: macs3 callpeak -t $chip -f BAMPE -g mm10 -n $out -p 1e-9 --keep-dup all --slocal 1000 --shift 100 --nolambda. Peaks overlapping blacklisted genomic regions were excluded from downstream analysis. Read-depth normalized bedGraph coverage files were converted to bigWig format using the bedGraphToBigWig utility.

For identifying differential SATB2 binding sites, we first merged bam files from five replicates (Bic-treated or NBQX-treated) and used the merged bam files as input files for *MACS3*. To detect gained SATB2 peaks, Bic-treated merged sample was considered as treatment and NBQX as control (Bic/NBQX). Conversely, for identifying lost SATB2 peaks, NBQX-treated merged sample was considered as treatment and Bic-treated as control (NBQX/Bic). Next, we used MAnorm to call differential and common peaks independently using the same merged bam files as input files. Peaks with M-values >0.3 were defined as Bic-specific, those with M-values <−0.3 as NBQX-specific, and the rest as common peaks. Final gained and lost peaks were obtained by intersecting *MACS3*-called gained/lost peaks with Bic-specific and NBQX-specific peak sets, respectively. To define invariant SATB2 peaks, the final sets of gained and lost peaks were subtracted from MAnorm-derived common peaks, and the remaining set of peaks was referred to as invariant peaks.

For visualization, merged coverage files were generated using the bamCoverage function from *deepTools* (*v3.5.6*) with the following parameters: --binSize 50 --normalizeUsing *RPGC* --effectiveGenomeSize *mm10*. Peak annotation was done by using ChIPseeker [42]. To determine the overlap between SATB2 peaks and additional TFs, SATB2 summits were extended ±250 bp. Peak overlaps were determined using bedtools/2.27.1 intersect bed allowing a minimum of 1 bp overlap. Average profiles were generated using deeptools function *computeMatrix*, 50 bp bin size.

Genome browser visualizations for specific regions of interest were generated using pyGenomeTracks (v3.5) [30]. For these plots, SATB2 occupancy from bigWig files, differential binding sites from narrowPeak files, chromatin interaction arcs derived from Hi-C data, and gene models from a reference annotation (GTF) were integrated. The layout, colours, and display parameters for all tracks were defined in a configuration file (tracks.ini), and pyGenomeTracks was then used to visualize the specified genomic coordinates.

### RNA-seq

#### RNA extraction and library preparation

Primary cortical neurons (1 × 10⁶) were washed twice with 1× PBS and collected in 1 ml of TRIzol (Invitrogen, #15596026). RNA was extracted using the PureLink™ RNA Micro Scale Kit (Invitrogen, #12183016) according to the manufacturer’s protocol. RNA quality was assessed using an Agilent TapeStation. Samples with a RIN score >8 were used for library preparation and sequencing.

For the 6 h Bic- and NBQX-treated samples, poly(A)-enriched libraries were prepared by Novogene and sequenced on the Illumina NovaSeq platform using 150 bp paired-end (PE) reads, with a sequencing depth of 35 million reads per replicate.

RNA-seq data from 1 h floxed and cKO Bic- and NBQX-treated samples have been deposited in GEO (GSE157375).

#### RNA-seq analysis

Raw reads were trimmed using *trimmomatic* (v0.39) [43] with the following command: java -jar trimmomatic-0.39.jar PE $reads1$reads2$out3.trimmed.gz $out4.gz $out5.trimmed.gz $out6.gz LEADING:3 TRAILING:3 MINLEN:36 SLIDINGWINDOW:4:15 -threads 2 ILLUMINACLIP:Trimmomatic-0.39/adapters/TruSeq3-PE.fa:2:30:10:2:True -summary $out3.stat.summary. Trimmed reads were mapped to mm10 using STAR (2.7.4a) [44]. The output bam files were sorted, indexed and flagstated. The *featureCounts* [45] package of subreads (v2.0.6) (subread/2.0.6/bin/featureCounts) was used to count reads to obtain read counts along genes for each sample. The count matrices were generated per time point (1 h and 6 h) and corrected for batch effect using RUVr (*k* = 3) function of *RUVseq* R package [46]. For the differential gene expression analysis, the *DESeq2* package was used to generate normalized counts [47]. After running a standard *DESeq2* pipeline, normalized counts were generated with the function *counts*. For differential gene expression, a threshold cutoff of adjusted *P*-value <.05 and basemean >30 was applied. Volcano plots were generated using the R package *EnhancedVolcano*. RRHO analysis was performed as previously described [48, 49].

### Functional enrichment analysis

Metascape [50] was used to identify overrepresented GO terms. As ontology sources, GO Molecular Functions, GO Biological Processes, and GO Cellular components were used. Terms with a *P*-value <.01, a minimum count of 3, and an enrichment factor >1.5 (the ratio between the observed counts and the counts expected by chance) were collected and grouped into clusters based on their membership similarities. *P-*values were calculated based on a cumulative hypergeometric distribution, and q-values were calculated using the Benjamini–Hochberg (BH) procedure for multiple testing. All genes in the genome were used as background except for GO enrichment analyses of differentially expressed genes (DEGs), where a custom background was used, consisting of mouse brain-expressed genes. GO enrichment analysis of gene sets above 3000 genes was performed by using clusterProfiler [51].

Fisher’s exact test was employed to test for significant overlaps between gene sets.

### Enrichment analysis of genomic regions

To determine the enrichment of previously annotated chromatin states (18-state model P0 forebrain, ENCFF758EGD) in SATB2 extended (±250 bp) peaks we used *GRanges* objects of the *GenomicRanges* package and *findOverlaps* function in R. Enrichment visualization was done using the *pheatmap* package in R. Additionally, we assessed spatial and local enrichments between sets of genomic regions using the *regioneR* package through permutation testing (*n* = 10 000 iterations). The *P*-value and local Z-score were derived from *overlapPermTest* function, while the significance threshold (α < 0.05) was applied during randomization.

### Analysis of Genomic Run-On Sequencing data

Published Genomic Run-On Sequencing (GRO-seq) data from murine primary cortical neurons [52] were analysed to assess promoter-proximal RNA Pol II pausing at genes mapped to invariant SATB2 peaks in the AD compartment. GRO-seq read coverage from untreated control neurons (0 min, designated CTRL) and neurons treated with 55 mM KCl for 60 min (KCl) was quantified at gene promoters and gene bodies. Promoter regions were defined as −100 bp to +200 bp relative to the TSS, and gene body regions as +400 bp to +800 bp downstream of the TSS. Pausing indices were calculated as the ratio of promoter-proximal read density to gene body read density. Read coverage was computed in non-overlapping 10 bp bins for both conditions.

### Statistical analysis

Data were obtained from independent biological replicates (cultured cortical neurons isolated from independent mouse litters, as described in the method details). Descriptions of data collection, data quantification, and statistical methods used to analyse each experiment are provided in the respective sections in the method details or in figure legends. Exact *P*-values and number of replicates (*n*) can be found in the main text and/or figure legends.

## Results

### SATB2 is required for neuronal activity-triggered chromatin compaction

To investigate how increased neuronal activity influences global chromatin architecture, chromatin accessibility, and gene expression in cortical neurons, we generated Hi-C contact maps, chromatin accessibility profiles and transcriptome data from primary cortical neurons that were either silenced for 16 h with the AMPA receptor antagonist NBQX or stimulated for 1 h with bicuculline (Bic). Bicuculline, a GABA*_A_* receptor antagonist, disinhibits cortical networks, leading to enhanced excitatory drive, increased activation of synaptic NMDA receptors, and a physiological increase in action potential firing [53]. To assess the role of SATB2, activity-induced transcriptomic and 3D epigenomic changes were compared between cortical neurons derived from Satb2^flx/flx^ mice (floxed) and Satb2 ^flx/flx^::*Nes-Cre* mice (cKO) (Supplementary Fig. S1A–G). In addition, activity-dependent transcriptomic responses were analysed in *AAV-Cre*-transduced floxed cultures, representing an acute *Satb2* deletion model (acute KO). To control for secondary effects of *Satb2* loss on chromatin accessibility changes, cKO neurons were further compared with “rescued cKO” neurons, acutely re-expressing SATB2 via transduction with *Satb2*-encoding AAVs (Supplementary Fig. S1A–G).

Cortical neurons derived from *Satb2* cKO mice and neurons with acute *in vitro Satb2* deletion exhibited similarly impaired activity-dependent transcriptomic responses at both 1 h and 6 h after treatment. Both up- and down-regulatory trajectories were affected. Upregulated genes in response to neuronal activation were enriched for pathways involved in synapse organization, neuron projection morphogenesis, and G-protein coupled receptor activity, whereas downregulated genes in the same response were enriched for general cell biological processes related to translation, ribonucleoprotein complex biogenesis, mRNA processing, and regulation of post-translational protein modification (Supplementary Fig. S2A–J).

**Figure 1.**
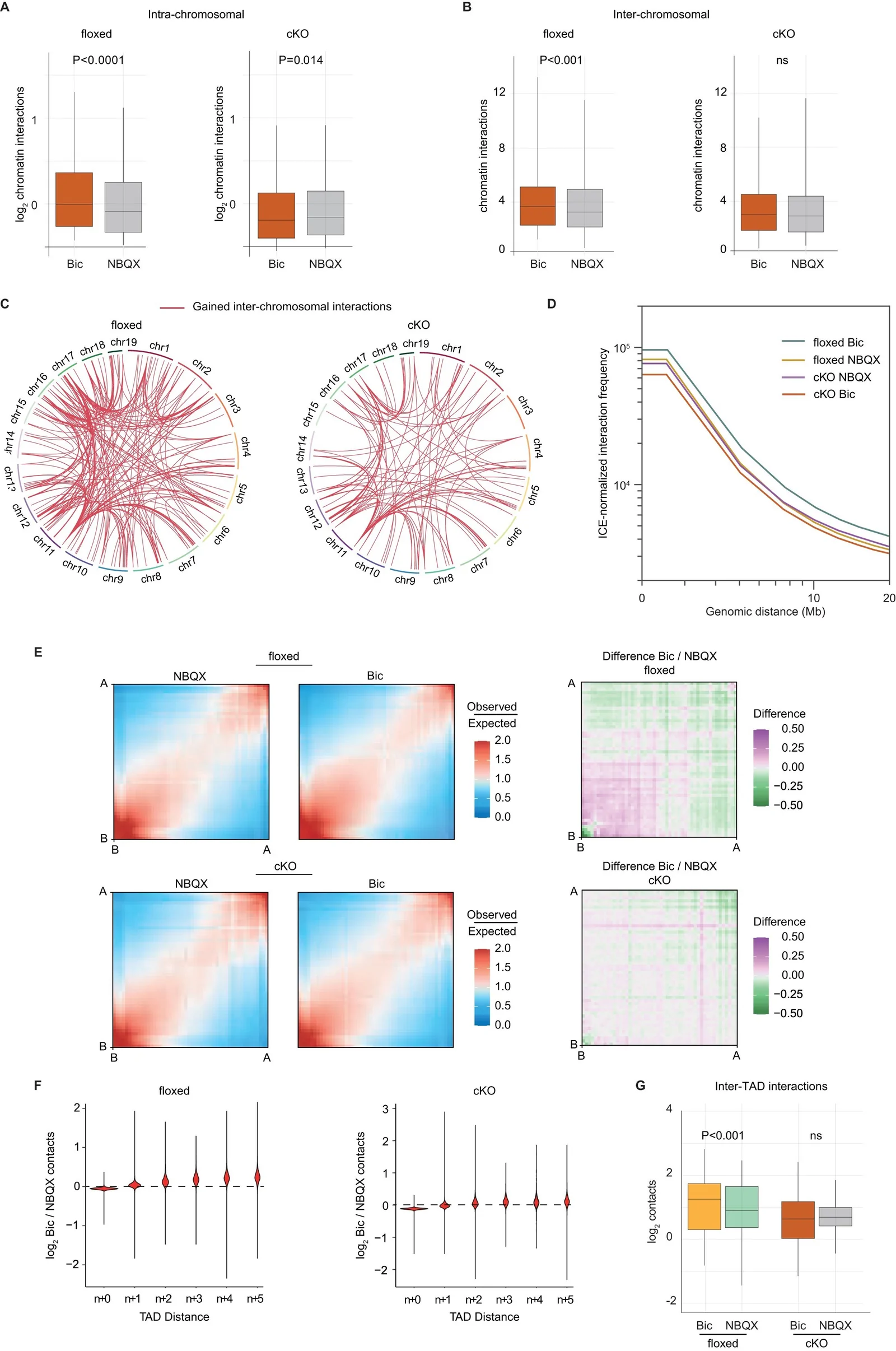
Global aggregate analyses of Hi-C contact matrices reveal increased chromatin interactions upon bicuculline-induced AP bursting in floxed but not *Satb2* cKO cortical neurons. Quantification of averaged intra-chromosomal (**A**) and inter-chromosomal (**B**) interactions at 100 kb resolution in Bic- versus NBQX-treated floxed (left) and cKO (right) cultures. Statistical significance was assessed using two-tailed Mann–Whitney tests. (**C**) Circos plots illustrating activity-induced gained inter-chromosomal interactions in floxed (left) and cKO (right) cultures. (**D**) Contact distance–decay plots at 100 kb resolution in Bic- versus NBQX-treated floxed and cKO cultures. (**E**) Saddle plots showing enhanced B–B interactions in active versus silenced floxed but not cKO cortical neurons. Shown are average O/E contact enrichment values between pairs of 100 kb bins, ordered by compartment signal strength (EV1). Differential saddle-plots (right) highlight increased and decreased interactions in Bic- versus NBQX-treated floxed but not cKO samples. (**F**) Differential TAD + N analysis comparing interaction frequencies within TADs and their first to fifth neighbouring TADs between Bic- and NBQX-treated floxed (left) and cKO (right) neurons. (**G**) Quantification of averaged inter-TAD interactions in Bic- versus NBQX-treated floxed and cKO cultures. Statistical significance was assessed using two-tailed Mann–Whitney tests.

In floxed cultures, Bic stimulation for 1 h increased both intra- and inter-chromosomal contacts (Fig. 1A–C). Decay plot analysis revealed that the increase in intra-chromosomal interactions spanned all genomic distances from 100 kb to 20 Mb (Fig. 1D). In contrast, in Bic- versus NBQX- treated *Satb2* cKO cultures, intra-chromosomal contacts were even modestly decreased. Inter-chromosomal interaction frequencies were not significantly altered (Fig. 1A–C). Decay plots of Bic- versus NBQX-treated cKO neurons also showed an opposite pattern compared with floxed neurons, with interaction frequencies decaying more rapidly across the same genomic distance range in active than in silenced neurons (Fig. 1D). Therefore, neuronal activity induces a rapid, SATB2-dependent increase in chromatin compaction across all genomic distance scales. This finding implicates multiple mechanisms operating across varying genomic distances. To dissect this further, we performed global aggregation analyses at different hierarchical levels of 3D genome organization.

Saddle plot analysis revealed that Bic treatment induces a global but non-uniform modulation of B–B compartment interactions in floxed neurons, with increased interactions across most B–B contacts and a decrease in the most B-like bins (lower-left corner of the saddle plot) (Fig. 1E). In cKO cultures, both the overall reinforcement of B–B interactions and the subset-specific decrease in B-B contacts were blunted. At the TAD level, floxed but not cKO neurons exhibited significantly increased inter-TAD interactions, between up to five neighbouring TADs, under active compared with silenced conditions (Fig. 1F and G). Thus, SATB2 is required for activity-dependent modulation of B–B compartmentalization and strengthening of inter-TAD contacts.

**Figure 2.**
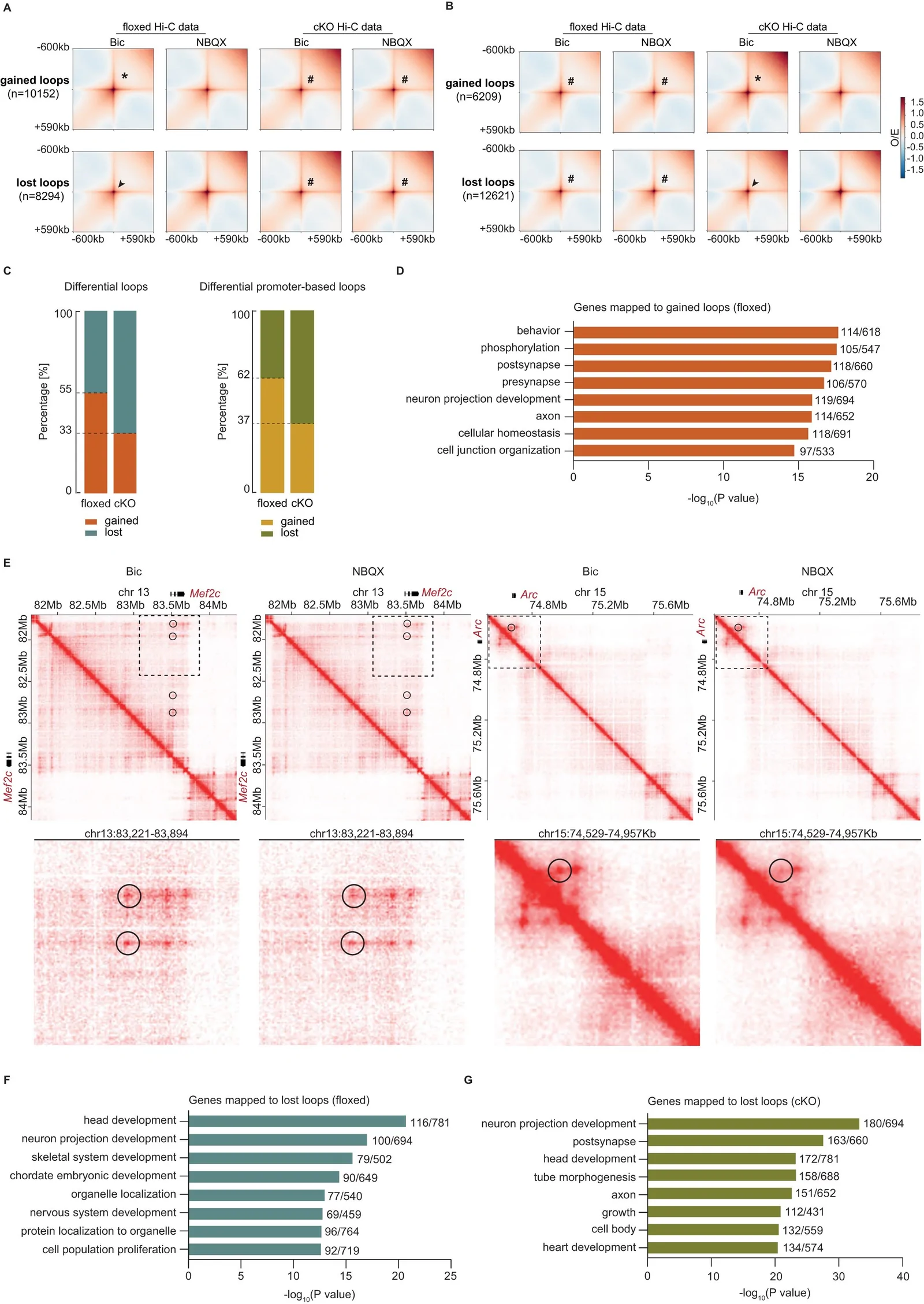
Activity-triggered chromatin loop reconfiguration is reversed in SATB2-deficient cortical neurons. Aggregate peak analyses centred at differential chromatin loops detected in floxed (**A**) and cKO (**B**) cultures, using Hi-C matrices at 10 kb resolution from Bic- and NBQX-treated samples. Asterisks denote increased chromatin interactions at gained loops, arrowheads indicate decreased interactions at lost loops, and hash symbols indicate no change between conditions. (**C**) Bar charts showing the proportions of differential loops and differential promoter-based loops in floxed and cKO cultures. (**D**) GO enrichment analysis of genes mapped to gained promoter-based loops in floxed neurons. The top eight most significant GO terms are shown. (**E**) Hi-C matrix snapshots of Bic- and NBQX-treated floxed samples illustrating gained promoter-based loops (outlined in black) at the *Mef2c* (left) and *Arc* (right) loci. Shown are ICE-normalized chromatin contacts at 10 kb (top) and 5 kb (bottom) resolution. Lower panels show zoomed-in views of the dashed regions. GO enrichment analysis of genes mapped to lost promoter-based loops in floxed (**F**) and cKO (**G**) neurons. The top eight most significant GO terms are shown.

At the chromatin loop level, neuronal activation induced both gained and lost interactions, reflecting *de novo* loop formation and loss, or changes in loop strength. In floxed neurons, more loops were gained than lost (10 152 versus 8294) upon Bic stimulation (Fig. 2A), suggesting that loop reconfiguration contributes to chromatin compaction in active neurons. In contrast, *Satb2* cKO neurons showed the opposite response to activation, with fewer gained (6209) and more lost loops (12 621) in active neurons, indicating an overall reduction in compaction at the loop level (Fig. 2B).

Given the established role of enhancer–promoter interactions in activity-dependent gene regulation, we next focused on promoter (P)-based loops, defined as loops with one or both anchors mapping to a promoter. Activity-induced changes in P-based loops mirrored the global trends observed for all loops. In floxed neurons, 4312 gained and 3389 lost P-based loops were identified, representing 42% and 41% of all gained and lost loops, respectively. By contrast, cKO neurons showed a predominance of loop loss, with 2543 gained versus 5523 lost P-based loops (41% and 43% of all gained and lost loops, respectively) (Fig. 2C). In floxed neurons, functional annotation revealed a striking divergence in the functional identity of genes mapped to gained versus lost P-based loops. Genes associated with gained loops were selectively enriched for synaptic functions (Fig. 2D and E), whereas those mapped to lost loops were enriched for brain development-related terms (Fig. 2F). This functional separation was absent in *Satb2* cKO neurons: genes mapped to gained loops showed no significant functional enrichment, while those linked to lost loops were instead enriched for both synaptic and neurodevelopmental categories (Fig. 2G).

We next tested whether the gene sets specifically enriched for synaptic GO terms—those linked to gained P-based loops in floxed neurons and lost P-based loops in cKO neurons—overlapped with previously identified ARGs [2] or DEGs identified in our cultures at 1 h and 6 h after Bic treatment (Supplementary Fig. S2A–H). In floxed neurons, genes mapped to P-based loops were enriched for ARGs [odds ratio (OR) = 1.8, *P* = .007] and DEGs (OR = 1.23 and 1.94, *P* = .003 and *P* < 1 × 10^–14^ at 1 h and 6 h, respectively). Similarly, in cKO neurons, genes mapped to lost P-based loops were enriched for ARGs (OR = 1.6, *P* = .0049) and DEGs (OR = 1.4 and 1.95, *P* = 2.36e-06 and *P* < 1e-14 at 1 h and 6 h, respectively; two-sided Fisher’s exact tests). These findings indicate that SATB2 is not required for activity-induced weakening of loops associated with developmental genes, which occurs in both floxed and cKO neurons. Instead, SATB2 is specifically required for the formation, strengthening, and maintenance of loops involving synaptic genes and ARGs. Importantly, SATB2 appears to carry out this regulatory role through two distinct sets of P-based differential loops: those gained in floxed neurons and those lost in cKO neurons. A particularly interesting subset of P-based loops are long-range loops (>700 kb), previously implicated in regulating LRGs [5]. We identified 1109 gained long-range P-based loops in floxed neurons, compared to only 542 in cKO neurons. Consistent with prior reports, genes mapped to gained long-range loops in floxed neurons were enriched for late activity-induced genes (upregulated at 6 h but not 1 h post-Bic stimulation; OR = 1.5, *P* = 3.67e-05, two-sided Fisher’s exact test). In contrast, no such enrichment was observed in cKO neurons, underscoring a critical role for SATB2-mediated long-range looping in LRG regulation. Taken together, our findings establish chromatin loop reconfiguration—encompassing both loop gains and losses—as an integral component of activity-induced chromatin interactions. A substantial fraction of these differential loops occurred at promoters and correlated with gene expression in floxed neurons, whereas in cKO neurons, gained P-based loops (both short- and long-range) lacked apparent functional relevance.

In conclusion, comparative Hi-C analysis of silenced and active cortical cultures revealed that neuronal stimulation within one hour drives increased contact frequency and chromatin compaction across multiple hierarchical levels of genome organization, including compartments, TADs, and loops. These rapid, activity-triggered 3D chromatin changes were absent—or even reversed—in *Satb2* cKO neurons.

### Activity-induced remodelling of open chromatin landscape is SATB2-dependent

To investigate whether SATB2 contributes to activity-induced remodelling of chromatin accessibility, we performed ATAC-seq in active and silenced cortical cultures. To control for potential secondary effects from long-term *Satb2* loss, we compared differential ATAC-seq signals across two complementary models. First, we compared *Satb2* Nes-Cre (cKO) cultures with floxed controls. We then compared cKO neurons with “rescued” neurons, in which recombinant *Satb2* was acutely re-expressed. The strong overlap, observed between both differential comparisons, argues against secondary effects resulting from prolonged SATB2 depletion in the cKO model (Supplementary Fig. S3A).

**Figure 3.**
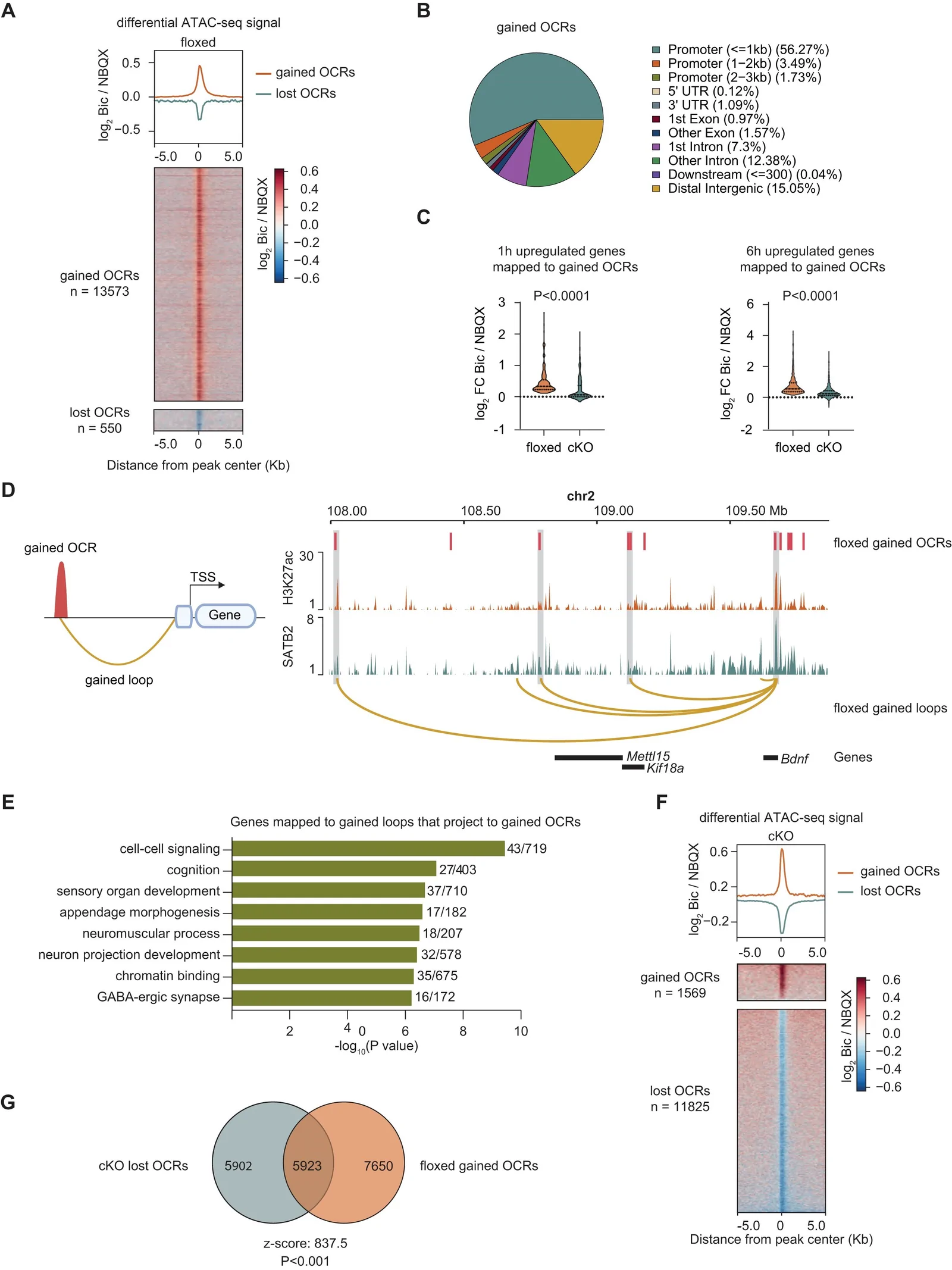
Activity-induced changes in the open chromatin landscape are SATB2-dependent. (**A**) Average profiles and heatmaps of differential ATAC-seq signal (log_2_ Bic/NBQX) plotted over gained and lost OCRs in floxed neurons. Data show merged signals from all replicates (*n* = 3). (**B**) Genomic annotation of gained OCRs in floxed cultures. (**C**) Activity-induced upregulation of genes mapped to gained OCRs at 1 h and 6 h post-Bic stimulation is impaired in cKO cultures (paired non-parametric Wilcoxon tests). (**D**) Schematic and genome browser tracks illustrating gained chromatin loops connecting gene promoters to gained OCRs. Browser tracks show gained OCRs (grey shading), aggregate H3K27ac CUT&Tag (*n*  =  2) signal, and SATB2 CUT&Tag signal (*n* = 10) in floxed cultures. Activity-induced gained loops connecting the *Bdnf* promoter to gained OCRs are shown as arcs. (**E**) GO enrichment analysis of genes connected to gained OCRs via gained loops. The top eight most significant GO terms are shown. (**F**) Average profiles and heatmaps of differential ATAC-seq signal (log_2_ Bic/NBQX) plotted over gained and lost OCRs in cKO neurons. Data show merged signals from all replicates (*n* = 3). (**G**) Venn diagram showing the overlap between gained OCRs in floxed neurons and lost OCRs in cKO neurons.

Differential analysis between Bic- and NBQX-treated neurons in each genotype identified three classes of ATAC-seq peaks: gained OCRs, defined as those with a log_2_ (Bic/NBQX) signal >0, lost OCRs (log_2_ (Bic/NBQX) <0); and invariant OCRs that remained unchanged upon stimulation (Supplementary Fig. S3B and C). We first studied the effect of neuronal activity in floxed cultures. Here, significantly more OCRs were gained than lost (13 573 versus 550) (Fig. 3A), consistent with previous reports of increased chromatin accessibility after neuronal stimulation [9, 54, 55]. Gained OCRs in our dataset overlapped with previously identified activity-inducible regulatory elements in the hippocampus [55], indicating shared chromatin responses across pyramidal neuron subtypes (Supplementary Fig. S3D). More than half of gained OCRs (56%) were promoter-associated (Fig. 3B), and motif analysis revealed enrichment for motifs for MEF2 family TFs, as well as FOS, JUNB, FOSL2, and NPAS4 (Supplementary Table S1). Genes mapped to these gained OCRs significantly overlapped with genes upregulated at 1 h and 6 h after Bic stimulation (OR = 2.03 and 3.98, respectively; *P* < 10⁻¹^4^), highlighting a strong link between increased chromatin accessibility and transcriptional activation (Fig. 3C and Supplementary Fig. S3F). In contrast, lost OCRs showed neither significant TF motif enrichment nor overlap with DEGs. Integrative analysis of Hi-C and ATAC-seq data further revealed that gained OCRs were enriched at anchors of gained chromatin loops (*z* = 59.3, *P* = .001). Among these, 686 gained OCRs overlapped with P-based loops. Genes associated with these loops were significantly enriched for synapse- and cognition-related GO terms and included both early and dPRGs such as *Egr3, Dusp5, Bdnf, Fosl2*, and *Nr4a3* (Fig. 3D and E), underscoring a coordinated gain in chromatin accessibility and looping during the regulation of activity-dependent genes.

In *Satb2* cKO neurons, activity-induced changes in chromatin accessibility were dramatically altered. Compared to floxed neurons, there were far more lost OCRs (11 825) than gained (1569) (Fig. 3F). Approximately half of the lost OCRs in cKO neurons overlapped with gained OCRs in floxed neurons (*z* = 837.5, *P* < .001) (Fig. 3G and Supplementary Fig. S3E), indicating that SATB2 loss not only prevents gains in accessibility but actively promotes its loss at specific regulatory elements. Moreover, coordination between chromatin looping and OCR gains was severely impaired in cKO neurons: only 41 gained OCRs were detected at the anchors of gained chromatin loops, and genes associated with these loops showed no significant GO term enrichment.

### Two types of SATB2 DNA binding sites exist in cortical neurons: activity-dependent and invariant

**Figure 4.**
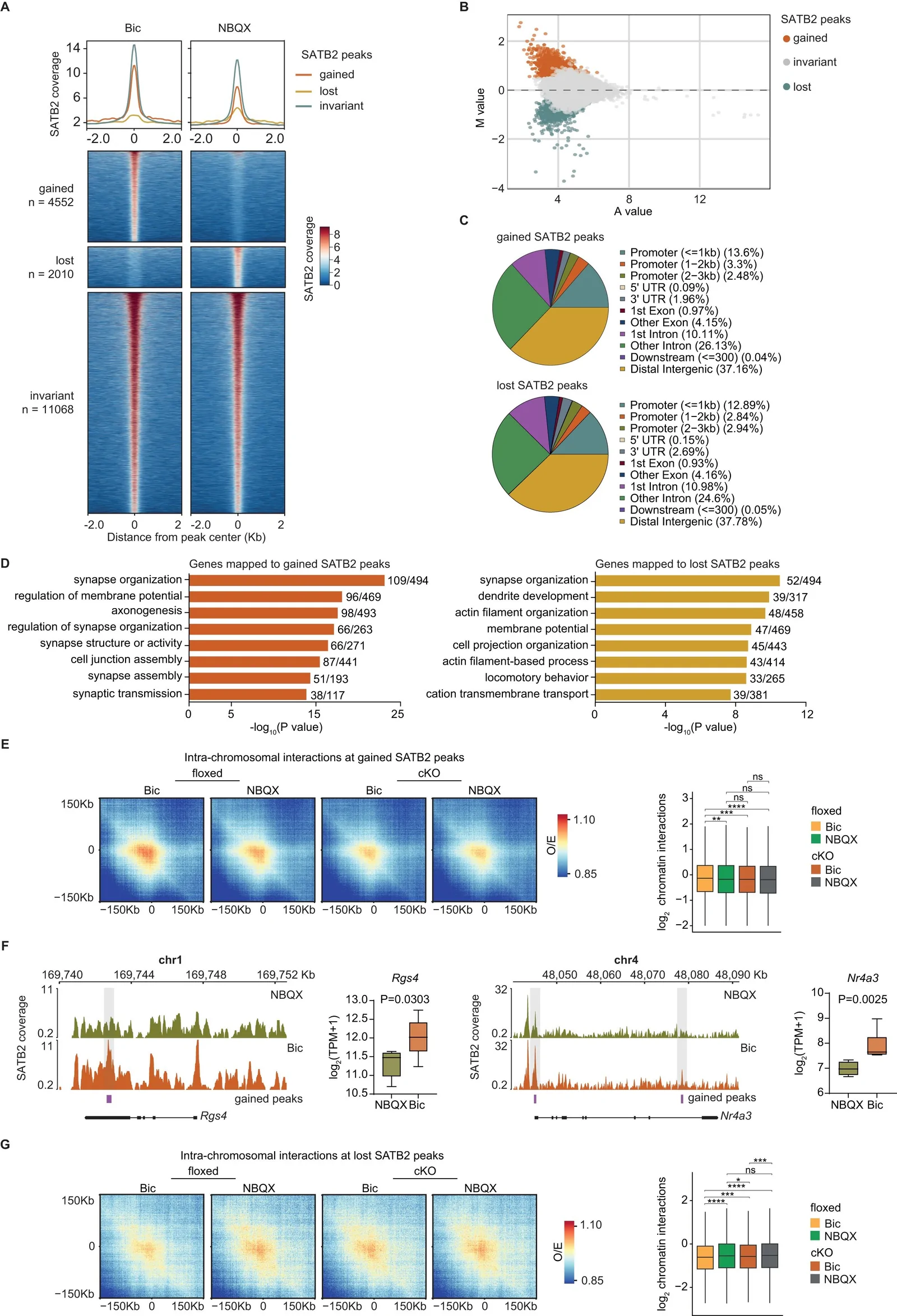
SATB2 binding to DNA is modulated by neuronal activity. (**A**) Average profiles and heatmaps of SATB2 CUT&Tag signal in Bic-treated (left) and NBQX-treated (right) cortical cultures at gained, lost, and invariant SATB2 binding sites. Each site is centred on the peak summit and extended ±500 bp. Data show RPGC-normalized merged signals from all replicates (*n* = 10). (**B**) MA plot showing log_2_-fold change (M value) in SATB2 CUT&Tag signal (normalized read density) in Bic- versus NBQX-treated samples as a function of average signal intensity (A value). Dark red and dark green points denote gained (log_2_-fold change > 0.3) and lost (log_2_-fold change <−0.3) SATB2 binding sites, respectively (*P* < .05). (**C**) Genomic annotation of gained (top) and lost SATB2 peaks (bottom). (**D**) GO enrichment analysis of genes mapped to gained (left) and lost (right) SATB2 peaks. The top eight most significant GO terms are shown. (**E**) Aggregate peak analysis of intra-chromosomal contacts centred on gained SATB2 peaks in Bic- and NBQX-treated cultures of both genotypes. Contact enrichment is shown as O/E interaction scores. Quantification of averaged intra-chromosomal interactions at 10 kb resolution is shown on the right (Kruskal–Wallis tests, *P < .05, **P < .01, ***P < .001, ^****^P < .0001, multiple testing correction by BH procedure). (**F**) Representative genome browser snapshots (left) and box plots (right) illustrating activity-induced gained SATB2 peaks and upregulation of nearby target genes following Bic treatment for 1 h (Mann–Whitney test). (**G**) Aggregate peak analysis of intra-chromosomal contacts centred on lost SATB2 peaks in Bic- and NBQX-treated cultures of both genotypes. Contact enrichment is shown as O/E interaction scores. Quantification of averaged intra-chromosomal interactions at 10 kb resolution is shown on the right (Kruskal–Wallis tests, *P < .05, **P < .01, ***P < .001, ^****^P < .0001, multiple testing correction by BH).

To test whether SATB2’s diverse effects on chromatin dynamics are linked to activity-dependent changes in its binding to DNA, we performed SATB2 CUT&Tag in silenced versus stimulated cortical cultures. Using two complementary statistical approaches, high-confidence differential and invariant SATB2 peaks were determined. We identified 4552 gained, 2010 lost, and 11 068 invariant peaks (Fig. 4A and B).

SATB2 binding sites (including invariant, gained, and lost peaks) were significantly enriched at anchors of activity-dependent differential loops (*z* = 32.23, *P* < .001) and showed weaker but still significant enrichment at invariant loop anchors (*z* = 13.7, *P* < .001). These results indicate that SATB2 preferentially occupies genomic regions that coincide with loop anchors, particularly those involved in activity-dependent reorganization.

Activity-dependent SATB2 peaks (gained and lost) were predominantly located in distal intergenic and intronic regions (Fig. 4C) and were significantly enriched for genic enhancers, as defined by ChromHMM annotations from neonatal forebrain signatures (Supplementary Fig. S4A). Lost peaks additionally showed enrichment within heterochromatic regions. Genes associated with differential SATB2 binding exhibited strong and selective enrichment for synaptic GO terms (Fig. 4D).

To assess the relationship between activity-dependent SATB2 binding and chromatin looping, we performed aggregate peak analysis. Upon stimulation, we observed increased intra-chromosomal contacts centred on gained SATB2 peaks in floxed but not cKO neurons (Fig. 4E). Consistently, gained SATB2 peaks significantly overlapped with genes mapped to gained chromatin loops (OR = 2.49, *P* < 1.0 × 10⁻¹⁵, two-sided Fisher’s exact test) and with genes upregulated at both 1 h and 6 h following stimulation (1 h: OR = 2.24, *P* = 1.07 × 10⁻¹¹; 6 h: OR = 2.06, *P* = .0014; two-sided Fisher’s exact test).

Representative examples of delayed primary response genes (dPRGs) with activity-dependent SATB2 recruitment following Bic stimulation together with increased gene expression after 1 h Bic stimulation are shown in Fig. 4F. Multiple additional dPRG loci exhibited increased chromatin interactions, gained SATB2 occupancy, and transcriptional activation upon stimulation (Supplementary Fig. S5), providing locus-level support for the genome-wide associations observed in Fig. 4E.

Lost SATB2 peaks showed reduced chromatin interactions upon Bic treatment in floxed neurons (Fig. 4G) and significantly overlapped with genes mapped to lost chromatin loops (OR = 2.64, *P* < 1.0 × 10⁻¹⁵, two-sided Fisher’s exact test). Together, these data indicate that activity-dependent SATB2 binding correlates with chromatin interactions and likely contributes to activity-induced chromatin loop reconfiguration at synaptic genes.

**Figure 5.**
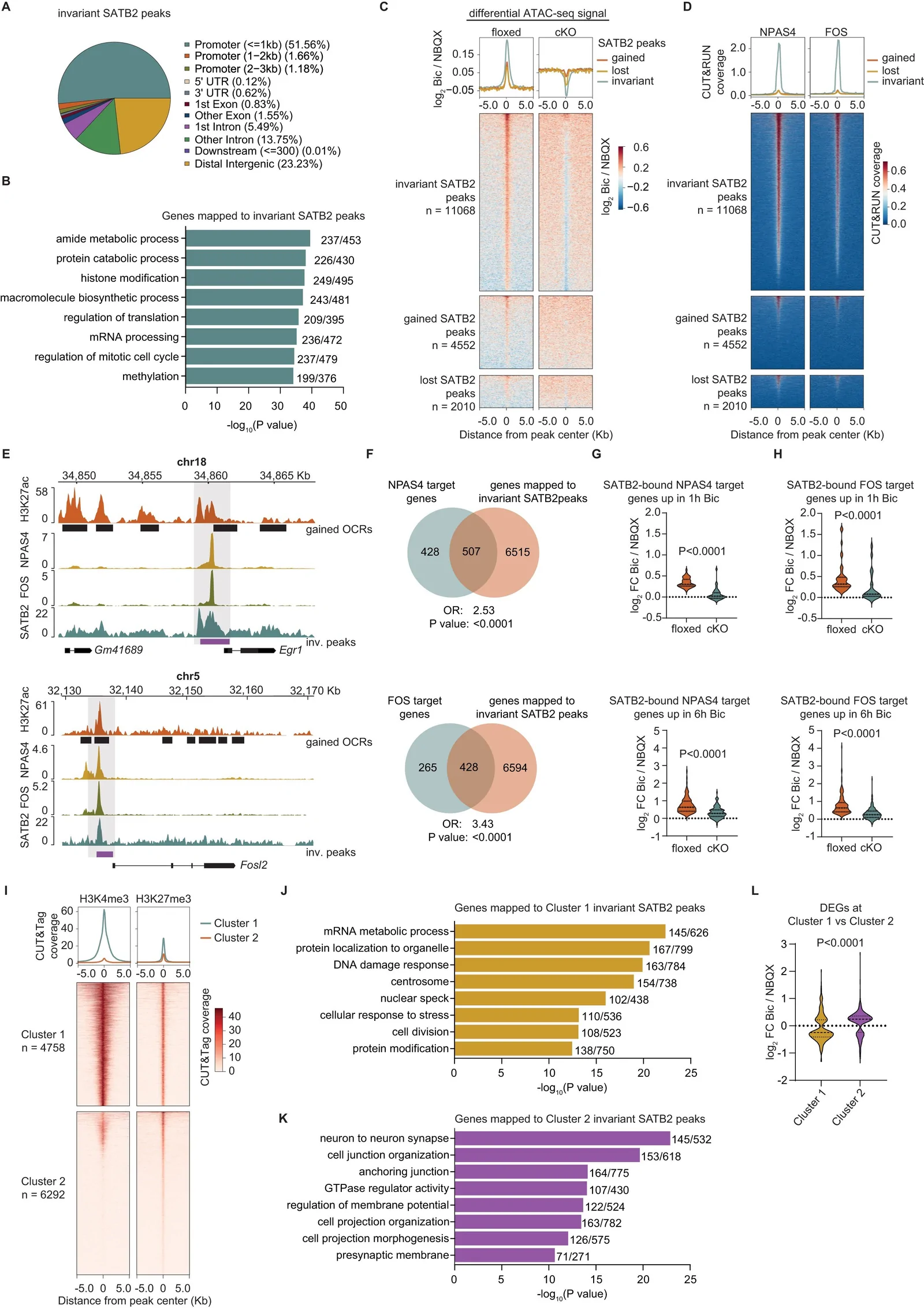
Invariant SATB2 peaks overlap with gained OCRs, activity-inducible TF binding sites, and bivalent promoters. (**A**) Genomic annotation of invariant SATB2 peaks. (**B**) GO enrichment analysis of genes associated with invariant SATB2 peaks. The top eight most significant GO terms are shown. (**C**) Average profiles and heatmaps of differential ATAC-seq signal (log_2_ Bic/NBQX) in floxed (left) and cKO (right) neurons plotted over invariant, gained, and lost SATB2 peaks. Each peak is centred on the summit and extended ±500 bp. Data show merged signals from all replicates (*n* = 3). (**D**) 1M-normalized CUT&RUN signals for NPAS4 and FOS (average profiles and heatmaps) from kainic acid-stimulated hippocampal tissue (2 h; [55]), plotted over gained, lost and invariant SATB2 peaks. (**E**) Genome browser tracks showing normalized CUT&Tag signals for H3K27ac and SATB2, CUT&RUN signals for NPAS4 and FOS (1M-normalized), gained OCRs, and invariant SATB2 peaks at the activity-inducible genes, *Fosl2* and *Egr1*. (**F**) Venn diagrams showing overlap between genes mapped to invariant SATB2 peaks and NPAS4 target genes (top; [55]) and FOS target genes (bottom; [56]). Activity-induced expression of SATB2-bound NPAS4 target genes (**G**) and FOS target genes (**H**) is impaired in cKO cultures at both 1 h and 6 h post-Bic stimulation (paired non-parametric Wilcoxon tests). (**I**) Average profiles and heatmaps of H3K4me3 and H3K27me3 CUT&Tag signals plotted over invariant SATB2 peaks and clustered by k-means (*k* = 2). GO enrichment analysis of the genes associated with Cluster 1 (**J**) and Cluster 2 (**K**) invariant SATB2 peaks. The top eight most significant GO terms are shown. (**L**) Violin plot illustrating log_2_-fold change in expression of DEGs identified in floxed neurons at 1 h following Bic treatment, stratified by association with Cluster 1 or Cluster 2 invariant SATB2 peaks (paired non-parametric Wilcoxon tests).

In contrast to differential peaks, invariant SATB2 peaks were predominantly promoter-associated (Fig. 5A) and enriched for active and bivalent promoter signatures, as well as proximal enhancers (Supplementary Fig. S4A). Genes associated with invariant peaks showed enrichment for metabolic and homeostatic processes and only modest enrichment for synaptic functions (Fig. 5B). ATAC-seq data in floxed neurons revealed increased chromatin accessibility at invariant but not differential SATB2 peaks, following neuronal stimulation (Fig. 5C and Supplementary Fig. S4B). Invariant peaks showed a strong and significant overlap with activity-gained OCRs (*z* = 337.47, *P* < .001, Supplementary Fig. S4C). Remarkably, the opposite pattern was observed in *Satb2* cKO cultures, where regions with invariant peaks identified in floxed neurons exhibited decreased accessibility upon stimulation, indicating that SATB2 is required for activity-induced chromatin remodelling at these sites (Fig. 5C).

Invariant, but not differential peaks, also co-localized with binding sites of activity-inducible TFs, including NPAS4 and FOS [55] (*z* = 372.85 and 259.25, respectively, *P* < .001; Fig. 5D and E, and Supplementary Fig. S4D). Genes associated with invariant SATB2 peaks were significantly enriched among NPAS4 and FOS target genes (Fig. 5F), and activity-induced expression of these targets [55, 56] was impaired in *Satb2* cKO neurons both 1 h and 6 h post-stimulation (Fig. 5G and H). We also noted that this impairment extended to all NPAS4 and FOS target genes, regardless of SATB2 binding to the genes, suggesting that SATB2 plays a broader permissive role in NPAS4- and FOS-mediated transcriptional responses to neuronal activity (Supplementary Fig. S4E and F).

To further dissect invariant SATB2 peaks, we examined their associated histone modification profiles. Integration of H3K4me3 and H3K27me3 CUT&Tag data from primary cortical neurons identified a subset of invariant SATB2 peaks corresponding to bivalent promoters (Cluster 1) (Fig. 5I). Genes associated with Cluster 1 peaks were enriched for GO terms related to mRNA and DNA metabolic processes, protein ubiquitination, and DNA damage response, whereas genes linked to non-bivalent invariant peaks (Cluster 2) were predominantly associated with synaptic functions (Fig. 5J and K). Cluster 1 genes were significantly enriched for genes downregulated 1 h following Bic application (OR = 1.56, *P *= 1.22903E-05, two-sided Fisher’s exact test), whereas Cluster 2 genes were enriched for genes upregulated at the same time point (OR = 2.14, *P* = 1.03E-11, two-sided Fisher’s exact test). Consistent with this, DEGs mapped to Cluster 1 peaks exhibited significantly stronger down-regulation compared to genes mapped to Cluster 2 peaks, as reflected by a downward shift in log_2_-fold-change distributions from more positive (Cluster 2) to more negative (Cluster 1) values (Fig. 5L). NPAS4 and FOS occupancy also differed between the two clusters: NPAS4 peaks were more strongly enriched at Cluster 1 compared to Cluster 2 SATB2 invariant peaks (*z* = 304.4 versus 237.62, *P* < .001), whereas FOS peaks showed the opposite pattern (*z* = 108.4 versus 246.2, *P* < .001). Cluster 1 peaks also showed stronger SATB2 and NPAS4 binding strength compared to Cluster 2 (Supplementary Fig. S4G).

In summary, cortical neurons harbour two classes of SATB2 binding sites, activity-dependent and invariant, each associated with distinct gene programs. Activity-dependent SATB2 peaks are predominantly associated with synaptic genes and contribute to activity-induced chromatin reconfiguration, whereas invariant peaks overlap with activity-gained OCRs and are associated with down-regulation of metabolic genes.

### The formation of a new activity-dependent compartment and TAD reorganization are SATB2-dependent

Previous studies have shown that neuronal activity can induce dynamic A/B compartment switches both *in vitro* and *in vivo* [9–11]. Given the observed down-regulation of metabolic genes associated with SATB2-bound bivalent promoters, we asked whether this repression was linked to a SATB2-dependent A-to-B compartment switch. Differential A/B compartment mapping revealed that Bic treatment triggered compartment switching, which is strongly dependent on SATB2 (Supplementary Fig. S6A and B). However, neither compartment switching nor changes in compartment strength correlated with gene expression changes of genes inside the differential compartment at 1 h or 6 h after stimulation (Supplementary Fig. S6C).

**Figure 6.**
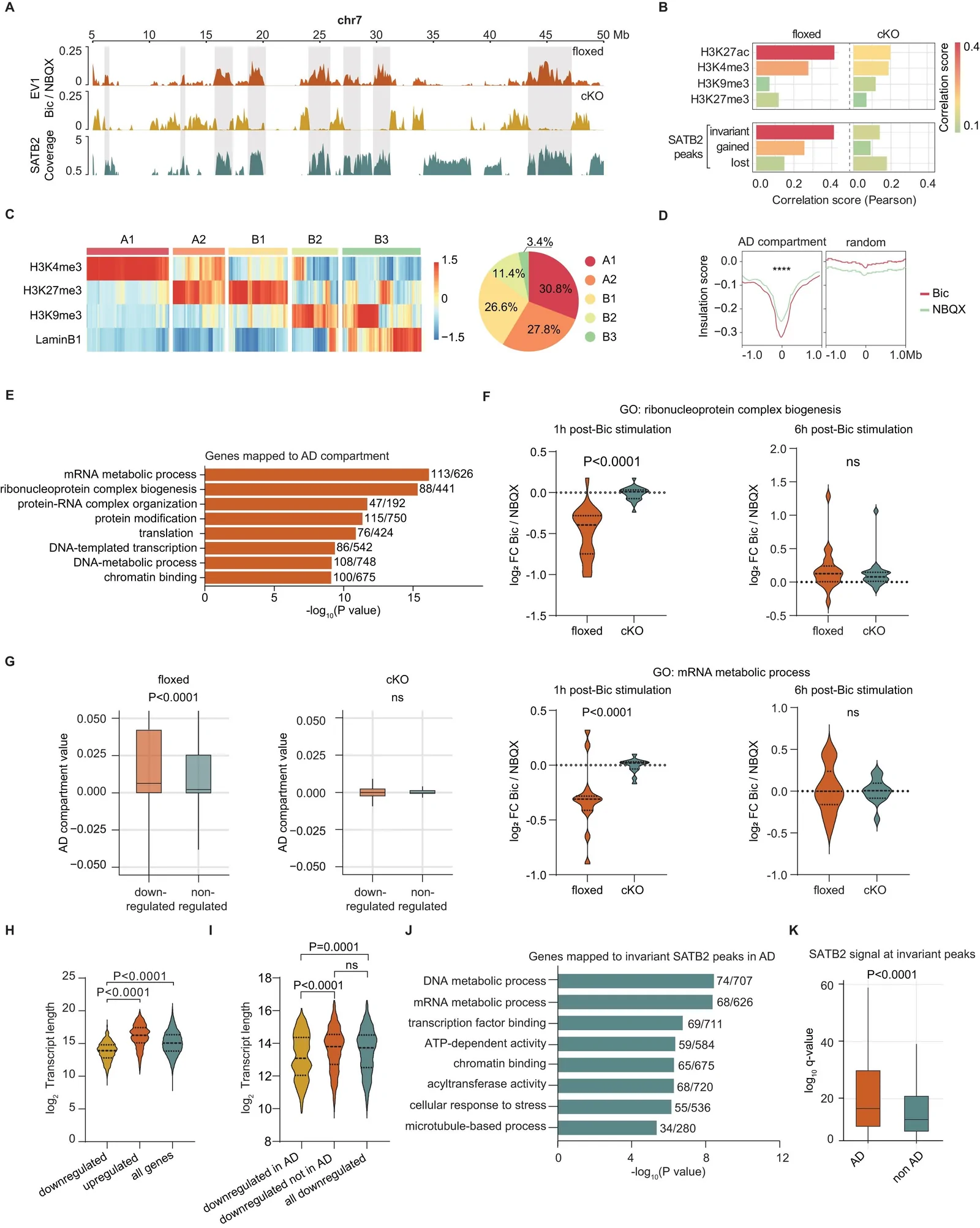
Neuronal activity triggers the formation of a novel chromatin compartment (AD compartment) in a SATB2-dependent manner. (**A**) Genome browser tracks of EV1 computed on differential (Bic/NBQX) Hi-C matrices in floxed (top) and *Satb2* cKO (bottom) cortical cultures. Shaded regions indicate loci assigned to the AD compartment in floxed but not in cKO neurons. The bottom-most track represents the SATB2 CUT&Tag signal in floxed neurons. (**B**) Chromatin features of AD compartment loci in floxed (left) and cKO (right) neurons. Pearson correlation coefficients were calculated on 100 kb resolution between AD compartment signal (EV1) and histone mark coverage (top) or SATB2 peaks (bottom). (**C**) Sub-compartment analysis showing the epigenetic composition of the AD compartment. Left: Heatmap displaying *k*-means clustering (*k* = 5) of the neuronal genome into five subcompartments (A1, A2, B1, B2, B3) based on normalized signals for H3K4me3, H3K27me3, H3K9me3, and Lamin B1. Right: Pie chart showing the proportional composition of AD compartment bins across these five sub-compartments, demonstrating an enrichment in active (A1) and poised (A2, B1) chromatin states. (**D**) Aggregate insulation score profiles at AD compartment boundaries (left) and matched random loci (right) in NBQX- (green) and Bic-treated (red) floxed neurons. AD compartment boundaries exhibit a strong insulation dip that intensifies with neuronal stimulation, a feature not observed at random loci (paired non-parametric Wilcoxon test, ^****^P = 2.2e-16). (**E**) GO enrichment analysis of genes mapped to the AD compartment in floxed neurons. The top eight most significant GO terms are shown. (**F**) Box plots showing expression changes for regulated genes (at 1 h post-stimulation) belonging to two representative GO categories from (**E**). The plots compare expression at 1 h (left) versus 6 h (right), demonstrating a transient downregulation in floxed cultures that is impaired in cKO neurons (paired non-parametric Wilcoxon tests). (**G**) Box plots showing the AD compartment signal (EV1) at downregulated versus non-regulated gene loci in floxed (left) and cKO (right) neurons (permutation test, *n* = 10 000). (**H**) Box plots comparing transcript lengths of downregulated, upregulated, and non-regulated genes at 1h post-stimulation (Kruskal–Wallis test). (**I**) Box plots comparing transcript lengths for downregulated genes (at 1h) located inside versus outside the AD compartment, relative to all downregulated genes (Kruskal–Wallis test). (**J**) GO enrichment analysis of genes mapped to SATB2 invariant peaks in the AD compartment. The top eight most significant GO terms are shown. (**K**) Box plot comparing SATB2 binding strength (CUT&Tag signal) at invariant peaks located inside versus outside the AD compartment (Mann–Whitney U test).

We therefore tested whether increased neuronal activity induces a novel chromatin compartment, analogous to the D compartment described following DNA double-strand break induction [57]. To this end, we applied PCA to differential Hi-C maps of Bic- versus NBQX-treated neurons on a per-chromosome basis. Positive values of the EV1 defined an activity-dependent chromatin compartment, which we termed the activity-dependent (AD) compartment (Fig. 6A and Supplementary Fig. S7A). For subsequent analyses, we applied an EV1 threshold of >0.05 to select chromatin domains and loci within the AD compartment exhibiting the strongest interaction frequency in response to neuronal stimulation; this threshold corresponds to the 75th percentile of positive AD compartment values assigned to all mouse promoters. To confirm that the AD compartment reflects a specific biological response and not a technical artifact arising from the comparison of any two Hi-C datasets, we performed the same analysis on differential maps generated from biological replicates of identical treatment condition (a pool of three versus another three replicates from the Bic condition and corresponding two pools for the NBQX condition). This control yielded a near-complete absence of bins surpassing the EV1 >0.05 threshold (Supplementary Fig. S7B and C), demonstrating that the AD compartment is a genuine feature of contrasting cellular states.

To characterize the epigenetic context of the AD compartment, we first broadly assessed its relationship with key chromatin features. This analysis confirmed that AD loci were enriched for active chromatin marks while being depleted of classical repressive marks (Fig. 6B). To dissect the epigenetic composition of the AD compartment at higher resolution, we next stratified the genome into five sub-compartments (A1-B3) by k-means clustering based on published (H3K4me3, H3K9me3, and Lamin B1) and newly generated (H3K27me3) epigenetic data. This method defined sub-compartments with distinct signatures, including active (A1), poised/bivalent (A2), and repressive states (B1–B3) (Fig. 6C). Mapping the AD compartment onto this refined epigenetic landscape showed it was predominantly composed of A1 (30.8%), A2 (27.8%), and B1 (26.6%) sub-compartments. This composition, particularly the strong enrichment in poised A2 and B1 states, indicates that AD regions are primed for rapid regulatory changes.

Finally, to validate the AD compartment using an orthogonal method, we performed insulation score analysis [58]. In floxed neurons, the boundaries of AD regions showed a clear local insulation minimum that deepened significantly upon Bic stimulation, consistent with activity-driven strengthening of their borders (Fig. 6D). This effect was specific, as a control set of matched random genomic bins showed neither insulation nor activity-dependent changes (Fig. 6D).

Notably, these structurally dynamic AD loci were strongly enriched for SATB2 binding sites, particularly invariant peaks (Fig. 6B), pointing to a direct role for SATB2 in orchestrating this reorganization. Consistent with this, AD compartment formation was severely impaired in *Satb2* cKO neurons: we identified 1 165 AD bins in floxed neurons compared to only 649 in cKO neurons, with 84% of the floxed AD bins being lost upon *Satb2* deletion (Supplementary Fig. S7A). The residual AD-like loci in cKO neurons were structurally compromised; their boundaries displayed weak baseline insulation and a complete lack of activity-dependent strengthening (Supplementary Fig. S7D). These loci also lacked a common biological identity, as their associated genes failed to show any significant GO term enrichment (Supplementary Table S2). Collectively, these deficits demonstrate that SATB2 is indispensable for assembling a functionally meaningful AD compartment and for orchestrating this architectural response to neuronal stimulation.

Functionally, genes within the floxed AD compartment showed strong enrichment for metabolic GO terms (Fig. 6E and Supplementary Table S2). DEGs assigned to the top enriched GO terms of this gene set were typically downregulated at 1 h, but not at 6 h, following Bic stimulation (Fig. 6F). Downregulated genes at 1 h post-stimulation were significantly more likely to reside in the AD compartment than non-regulated genes (OR = 1.853, *P* = 2.29e-9, Fisher’s exact test) and, as a group, exhibited stronger AD compartment signals than non-regulated genes (Fig. 6G and Supplementary Fig. S8A). The transient downregulation of these AD-associated DEGs was severely impaired in both acute and conditional *Satb2* deletion models (Supplementary Fig. S8B).

Transient downregulation of short metabolic transcripts has been proposed as a mechanism to prioritize translation of longer synaptic gene transcripts in response to neuronal activation [59, 60]. Indeed, Bic-downregulated genes were generally shorter than upregulated genes (Fig. 6H) and enriched for metabolic functions (Supplementary Fig. S2G). Notably, among the downregulated gene pool, those located within the AD compartment were significantly shorter than those outside of it (Fig. 6I and Supplementary Fig. S8C). Together, these results suggest that SATB2-dependent AD compartment formation mediates a coordinated, transient downregulation of short metabolic transcripts, thereby facilitating preferential translation of long synaptic genes during neuronal activation.

To gain further mechanistic insights into gene sequestration within the AD compartment, we next examined whether it correlates with SATB2 binding. We functionally annotated invariant and differential SATB2 peaks located within the AD compartment. Genes associated with invariant SATB2 peaks within AD were strongly enriched for DNA and mRNA metabolic processes, RNA splicing, protein ubiquitination, and TF binding, whereas genes associated with differential SATB2 peaks showed no significant GO term enrichment (Fig. 6J). Invariant SATB2 peaks within AD exhibited stronger SATB2 binding than invariant peaks outside AD (Fig. 6K) and extensively overlapped with Cluster 1 bivalent SATB2 peaks described earlier in Fig. 5I (OR = 23.74, *P* < 1e-14, two-sided Fisher’s exact test), linking them directly to poised chromatin. Using published GRO-seq data as a proxy for nascent transcription before and 1 h after KCl stimulation [52], we observed Pol II pausing at promoters of these genes (Supplementary Fig. S8D). This pausing occurred despite chromatin accessibility being unchanged or even slightly increased upon stimulation (Supplementary Fig. S8E), suggesting that gene sequestration into the AD compartment imposes a post-initiation transcriptional block.

**Figure 7.**
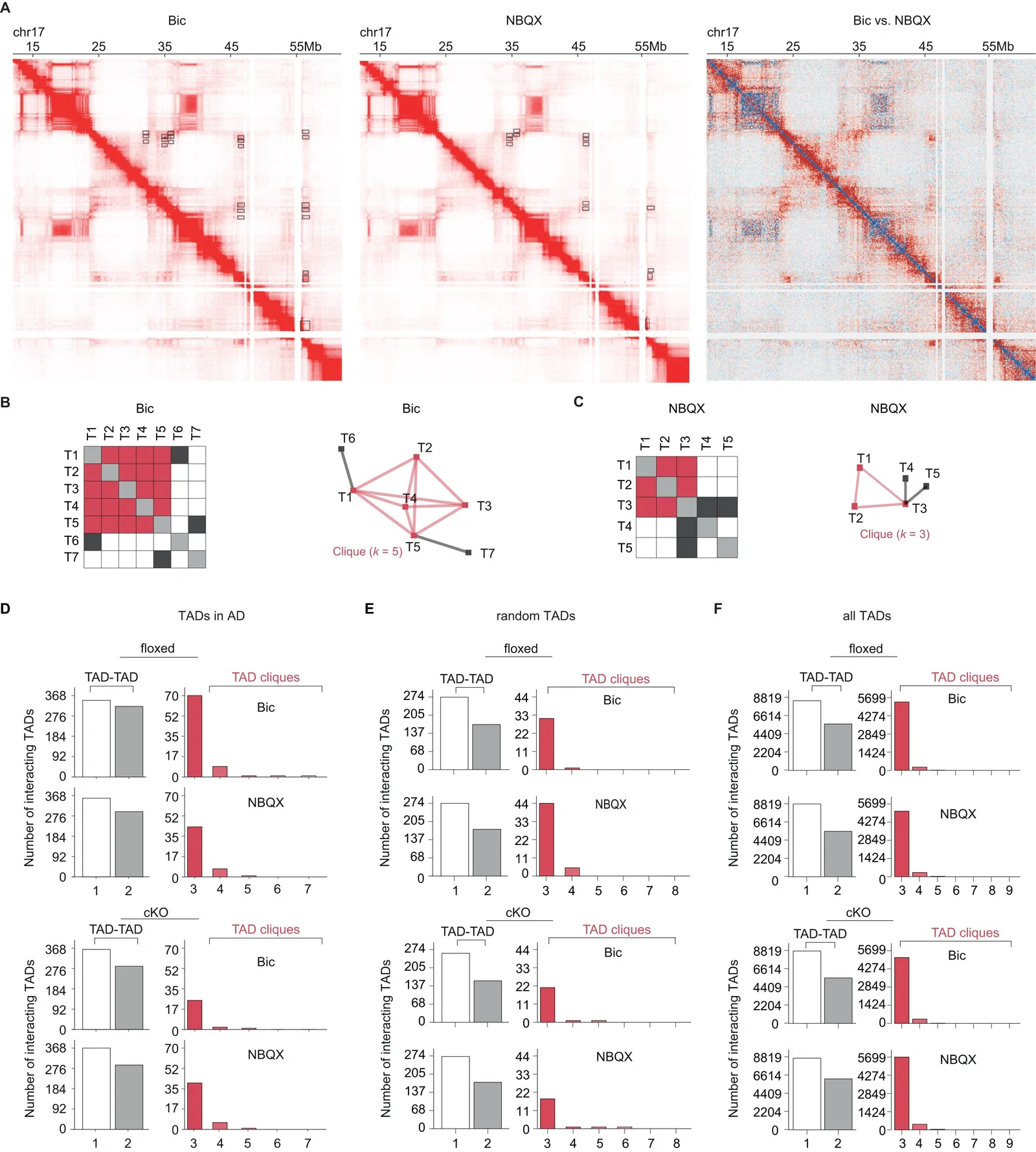
SATB2 drives activity-dependent clustering of its target TADs within the AD compartment. (**A**) Hi-C interaction matrices showing chromatin contact frequencies on a representative region of chromosome 17 in floxed neurons. Matrices are shown for Bic-treated (left) and NBQX-treated (middle) cultures. Black boxes highlight examples of TAD–TAD interactions that form cliques. (Right) The corresponding differential Hi-C matrix (Bic versus NBQX), where red indicates increased and blue indicates decreased interactions upon stimulation. (**B**) Incidence matrix and corresponding graph representation illustrating TAD clique formation in the Bic-treated condition for the region shown in (**A**). In the incidence matrix, red cells mark the significant interactions forming the clique. In the graph, nodes represent TADs and red edges denote the mutual interactions that constitute the highly connected structure (*k* = 5). (**C**) Incidence matrix and graph representation as in (**B**) for the NBQX-treated condition, showing a smaller clique (*k* = 3). (**D**) Bar plots quantifying total TAD–TAD interactions (left sub-panels, grey bars) and the number of TAD cliques grouped by size (right sub-panels, red bars). Clique size is defined by the number of member TADs (≥3, *x*-axis). This analysis focuses on the experimental set of TADs containing invariant SATB2 peaks within the AD compartment, comparing floxed (top) and *Satb2* cKO (bottom) neurons. Note that the activity-induced increase in clique formation is specific to floxed neurons. (**E**) Control analysis identical to (**D**) but performed on a randomly selected set of TADs matched for size and number. (**F**) Control analysis identical to (**D**) but performed on all TADs genome-wide.

To understand how SATB2 orchestrates the AD compartment, we next investigated the higher-order spatial organization centred around invariant SATB2 peaks within AD. To test whether SATB2-bound loci in the AD compartment cluster in nuclear space, we first analysed Hi-C data for long-range contacts between the TADs containing these loci. While mapping these regions identified a comparable number of TADs across all genotypes and conditions, their spatial behaviour was dramatically different. In floxed neurons, stimulation triggered a significant increase in the formation of higher-order TAD clusters, or “cliques” (Fig. 7A–D) [61]. Critically, quantification showed this activity-dependent clustering was entirely absent in *Satb2* cKO cultures (Fig. 7D). Control analyses using randomly selected TADs or genome-wide TAD sets showed no such changes, confirming that the activity-dependent clique formation is a specific feature of SATB2-bound AD compartment TADs (Fig. 7E and F).

**Figure 8.**
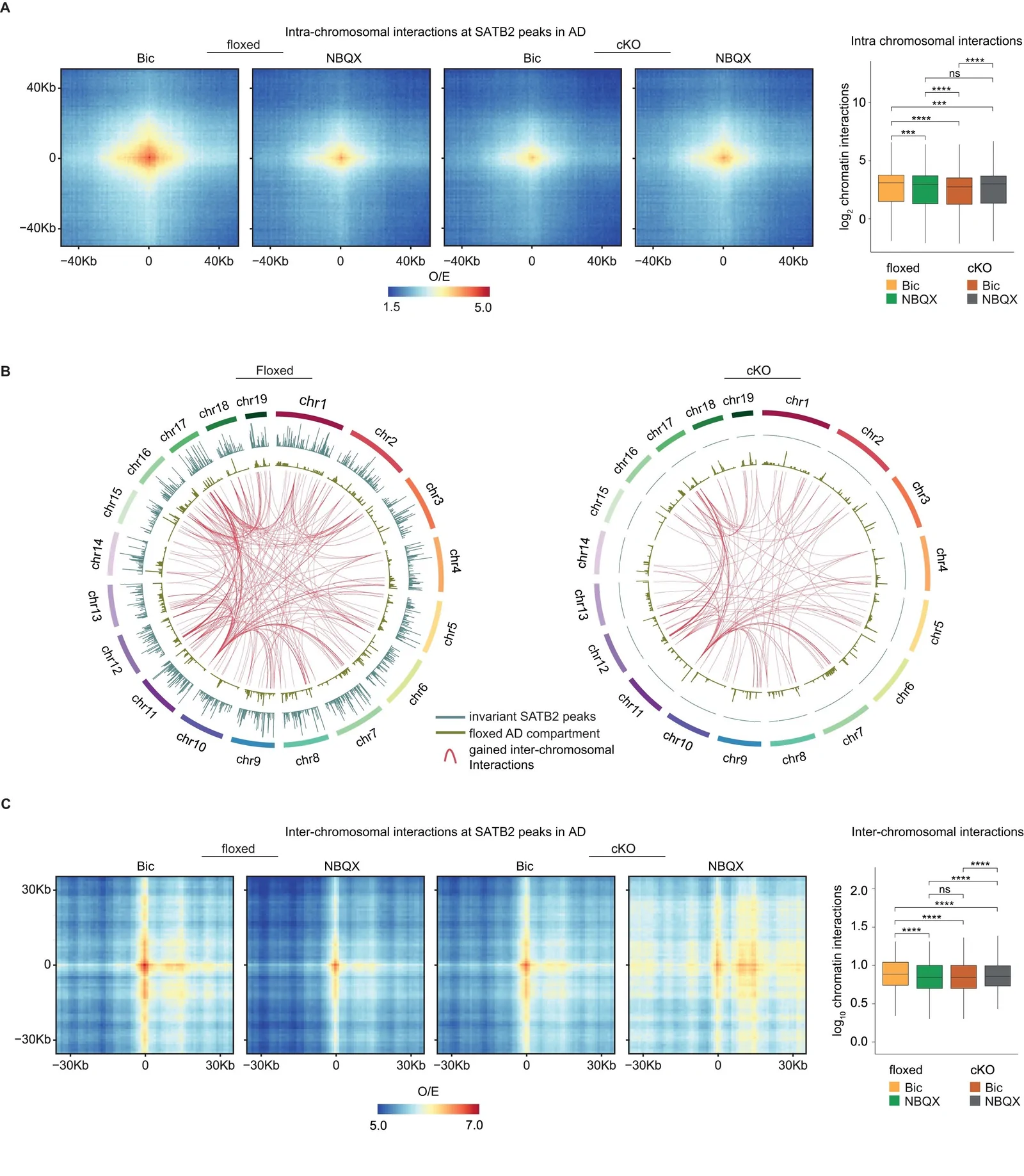
SATB2 orchestrates activity-dependent intra- and inter-chromosomal clustering of AD compartment loci. (**A**) Aggregate pile-up maps showing intra-chromosomal contact enrichment centred on invariant SATB2 peaks within the AD compartment. Maps display O/E scores for each genotype and treatment. The boxplot (right) quantifies log-transformed interaction strength, showing a significant increase upon stimulation in floxed but not in *Satb2* cKO neurons (Kruskal–Wallis test with BH correction, ***P < .001, ***P < .0001). (**B**) Genome-wide Circos plots visualizing gained inter-chromosomal contacts (red arcs) in floxed (left) and *Satb2* cKO (right) neurons following stimulation. The outer ring displays invariant SATB2 peak locations (teal), and the inner ring shows the floxed AD compartment score (olive). Note the dramatic reduction of activity-induced contacts in *Satb2* cKO cultures. (**C**) Aggregate pile-up maps of inter-chromosomal contacts centred on invariant SATB2 peaks within the AD compartment, showing O/E scores. The boxplot (right) quantifies log-transformed interaction strength, demonstrating that neuronal stimulation significantly increases inter-chromosomal contact strength specifically at these peaks and only in floxed neurons (Kruskal–Wallis test with BH correction, ns, not significant ^****^P < .0001).

This activity-induced clustering occurred at multiple scales within the AD compartment—not only between entire TADs but also directly between invariant SATB2 peaks (Fig. 8A). This clustering was not observed at the same regions in the cKO or at matched random regions in floxed neurons (Supplementary Fig. S9A). This spatial reorganization extended even between chromosomes; genome-wide Circos plots show that activity-induced inter-chromosomal interactions were strongly enriched within the AD compartment (*z* = 28.418, *P* < .001), particularly at invariant SATB2 peaks (*z* = 16.017, *P* < .001) (Fig. 8B). To confirm that SATB2 directly mediates these contacts, we performed aggregate pile-up analysis of inter-chromosomal contacts centred on invariant SATB2 peaks within the AD compartment. Neuronal stimulation selectively increased contact strength centred at these peaks in floxed, but not cKO cultures. This strengthening effect was also not observed at matched random regions in floxed neurons (Fig. 8C and Supplementary Fig. S9B).

Together, these converging analyses demonstrate that SATB2 drives the nuclear clustering of TADs and metabolic genes associated with the AD compartment upon neuronal activation. This architectural remodelling is achieved through the potentiation of both long-range intra-chromosomal contacts leading to TAD clique formation and targeted inter-chromosomal interactions centred on invariant SATB2 peaks. Ultimately, this SATB2-dependent spatial reorganization highly correlates with the transient downregulation of the affected metabolic genes.

## Discussion

Our study uncovers a central role for SATB2 in coordinating multi-scale chromatin remodelling in response to neuronal activity in cortical neurons. SATB2 drives formation and loss of chromatin contacts enabling dynamic transitions between transcriptionally permissive and repressive chromatin states upon activation. Loss of SATB2 disrupts this hierarchical coordination: activity-dependent chromatin accessibility, loop formation, long-range TAD–TAD interactions, and AD-compartment formation are all attenuated and correlated to blunted transcriptional responses. These findings highlight a model, in which SATB2 functions as a structural and regulatory hub, orchestrating dynamic changes in 3D genome architecture across all genome scales, from loop formation to TAD–TAD interactions and compartmentalization.

### The dynamic response of the 3D genome to an extracellular stimulus

So far, 3D chromatin reorganization has mostly been described in the context of cell differentiation [62–64], while the acute response of the neuronal 3D genome to physiological, extracellular stimulation has remained largely unexplored. By integrating Hi-C and chromatin accessibility maps from active versus silent primary cortical neurons, we provide the first genome-wide view of activity-induced 3D chromatin dynamics. Within just one hour of near-physiological AP bursting, we observe a global and widespread increase in chromatin interactions spanning both intra- and inter-chromosomal contacts and affecting all hierarchical levels, compartments, TADs and loops, resulting in overall chromatin compaction. The activity-driven strengthening of contacts across all distance scales detected by Hi-C is consistent with previous reports of large-scale chromatin condensation in stimulated neurons, observed microscopically [65]. These findings suggest that increased chromatin compaction, alongside enhanced chromatin accessibility [9, 66], constitutes a key component of the dynamic regulatory mechanisms underlying activity-induced transcription.

### The activity-dependent chromatin compartment regulates gene repression

In agreement with previous studies, we confirm that neuronal activity enhances chromatin accessibility and looping at synaptic genes, including ARGs [5, 13]. At these loci, we identify activity-induced strengthening of chromatin loops that connect gained OCRs to promoters, demonstrating coordination between accessibility and looping. Consistent with earlier reports [9–11], we also detect A/B compartment switching. However, unlike accessibility and looping, which closely track enhanced transcriptional responsiveness, A/B switches show little correlation with activity-induced gene expression. Instead, we uncover a previously unrecognized type of chromatin compartmentalization that exhibits a much stronger link to transcriptional changes.

We report the formation of a new chromatin sub-compartment, termed AD compartment, which together with long-range TAD–TAD interactions and increased inter-chromosomal interactions represents a highly coordinated chromatin response to neuronal stimulation. Mechanistically, our analyses reveal a two-tiered process driving this reorganization: the strengthening of local insulation at AD-compartment boundaries, which segregates them into discrete units, coupled with a concurrent increase in their preferential long-range interactions. Furthermore, our data establish this higher-order 3D genome restructuring as a novel candidate mechanism for activity-induced gene repression, the underpinnings of which so far have remained largely unexplored. We find that the AD compartment is enriched in active chromatin marks and SATB2 binding sites, yet, unexpectedly, it harbours a set of downregulated genes. These are primarily metabolic and housekeeping genes, which undergo a temporal downregulation at 1 h post-stimulation and recover expression at 6 h. While we did not directly test activity-dependent loss of H3K4me3 at bivalent promoters or reduction of histone acetylation, our ATAC-seq data reveal comparable chromatin accessibility at AD compartment-harboured loci under active and inactive conditions. Given the strong positive correlation between ATAC-seq and H3K27ac signals at active regulatory elements [67], a stimulation-induced reduction in histone acetylation at these loci is unlikely. By contrast, our analysis of published GRO-seq data [52] revealed RNA Pol II pausing at AD compartment–harboured loci 1 h after KCl stimulation. Because acute loss of H3K4me3 has been shown to promote RNA Pol II pausing and to reduce elongation rates [68], these findings suggest that activity-dependent loss of H3K4me3 at bivalent promoters is likely to underlie the observed transient repression. Furthermore, the transient downregulation of metabolic and housekeeping genes, which accompanies the well-described induction of IEGs, correlates not only with targeting to the AD compartment but also with spatial clustering of the corresponding gene loci within the nuclear space. The latter is mediated by multi-level chromatin interactions, including intra- and inter-chromosomal contacts and TAD clique formation [61], suggesting a higher-order sequestration mechanism. Functionally, the transcriptional repression of this specialized group of genes appears to serve an important regulatory purpose. Given that the ribosomal protein and housekeeping genes primarily encode short transcripts [59], their downregulation should lead to temporal reduction in the availability of short transcripts, thus promoting the efficient translation of long synaptic gene transcripts. This mechanism aligns with prior models proposing post-transcriptional prioritization of neuronal gene expression [60] but it now places it within a 3D genome context.

### SATB2 as a master regulator of activity-dependent 3D chromatin dynamics

Using cortical cultures from SATB2-deficient neurons, we demonstrate a pivotal role for SATB2 in activity-induced 3D chromatin dynamics. SATB2 contributes to all major chromatin architectural consequences of neuronal activation, including increased chromatin interactions across hierarchical levels, enhanced chromatin accessibility, and the formation of the AD compartment. Notably, SATB2 loss does not merely abolish these processes but often leads to their inversion, suggesting a balancing or homeostatic role for SATB2 in chromatin reorganization. Although we are currently unable to provide a specific mechanistic explanation for this reversal effect, it might be explained by competing binding of chromatin regulators to DNA. The dynamic equilibrium between competing factors is a central principle of chromatin regulation and is supported by biochemical, genomic, and perturbation evidence. For example, numerous studies show antagonism between SWI/SNF and Polycomb [69]. Thus, one plausible explanation is that SATB2 functions as a scaffold for activity-dependent multiprotein complexes that coordinate 3D chromatin remodelling and transcriptional regulation. In the absence of SATB2, these complexes may fail to assemble properly, allowing competing architectural or regulatory factors (e.g. cohesin or alternative TFs) to be ectopically recruited to SATB2 target sites. Such redistribution could invert the normal activity-dependent chromatin response, giving rise to the observed reversal effects.

The finding that SATB2, as a single chromatin-binding protein, can exert seemingly opposing effects on chromatin configuration likely reflects the context-dependent nature of its regulatory output. SATB2 function could be shaped not only by chromatin environment and interaction partners [19, 70] but also by post-translational modifications [71] and nuclear positioning [70]. Importantly, SATB2 binding to DNA itself is dynamically regulated by neuronal activity. We identify both invariant and activity-dependent (differential) SATB2 peaks, which fulfil distinct regulatory roles. Invariant peaks dominate both in number and versatility: they decorate bivalent promoters of metabolic genes within the AD compartment, forming interaction hot spots that enable coordinated repression, while also occupying activity-induced regulatory elements where SATB2 appears to co-bind with TFs such as FOS and NPAS4 [54–56], thereby facilitating activity-dependent gene induction. In contrast, the differential SATB2 peaks display a more specialized function. They preferentially occur at synaptic genes, where they appear to mediate activity-dependent chromatin interactions.

Together, our findings identify SATB2 as a key orchestrator of spatial and functional genome segregation following neuronal activation, adaptively coupling transient repression of metabolic programs with induction of neuronal gene expression. More broadly, they establish SATB2 as a cell type-specific 3D genome organizer that reshapes nuclear architecture and chromatin topology in cortical neurons in response to acute neuronal stimulation. This mechanistic role aligns well with the cognitive deficits observed in murine SATB2 loss-of-function models [18, 72] and in patients with SATB2-associated syndrome [73, 74].

## Supplementary Material

gkag788_Supplemental_Files

## Data Availability

Original data generated in this study have been deposited at GEO and are available as the following GEO records: GSE307197 (CUT&Tag), GSE307098 (RNA-seq), GSE307096 (ATAC-seq), GSE307097 (Hi-C). Published datasets used in this study are: GSE222609, GSE157375, GSE175965, GSE163113, GSM5738208, GSM5738212, GSM5738214, GSM5343756, GSM5343757. Histone modification ChIP-seq data were downloaded from ENCODE (ENCFF160SCR, ENCSR093DWU, ENCSR094TTT). Study analysis pipelines and codes are available at the following GitHub repository: https://github.com/Ali-Mujahid/Satb2-3D-Genome-organization and Zenodo at DOI: 10.5281/zenodo.21534635.
