# Supplementary material for: Neuronal activity-driven 3D chromatin dynamics in cortical pyramidal neurons depend on SATB2
Source: Nucleic Acids Res. 2026 Aug 25;54(16):gkag788. doi: 10.1093/nar/gkag788 (PMC13504278; doi:10.1093/nar/gkag788)
Supplement: Supplementary file 1 [file gkag788_supplemental_files.zip › Supplementary_Data.pdf]

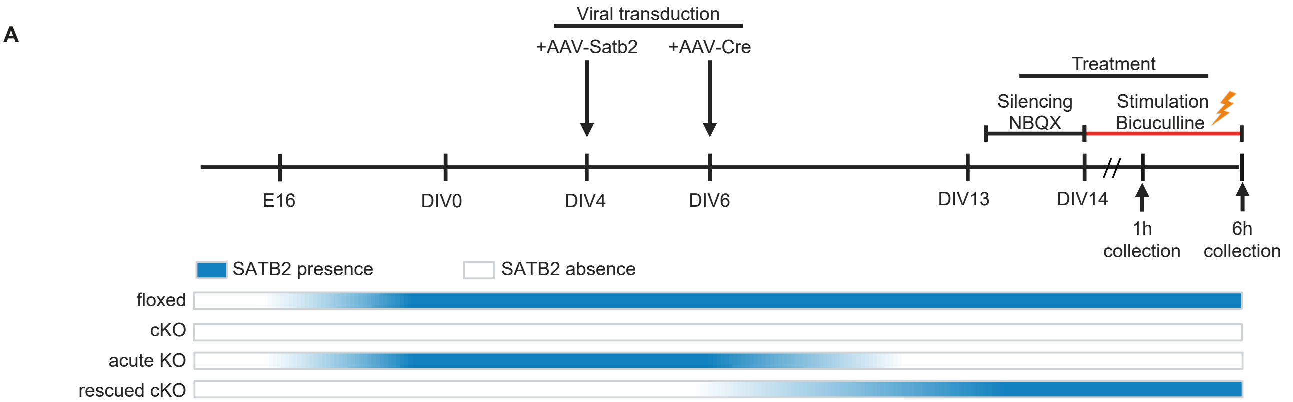

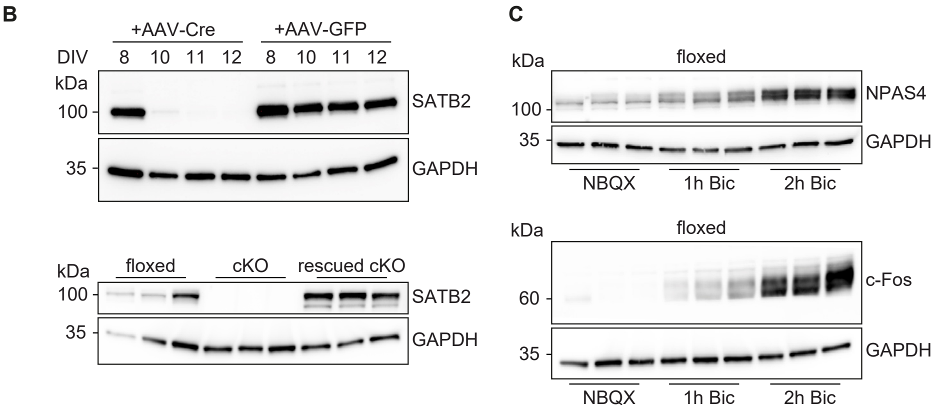

D

Expression levels of interneuron marker genes (log₂[TPM+1])

Gene	cKO	floxed	P value
Gad1	8.42	8.17	0.494
Gad2	6.82	6.81	0.781
Slc32a1	9.20	9.12	0.432
Sst	12.18	12.57	0.202
Vip	6.80	6.03	0.252
Reln	4.49	4.09	0.118
Lhx6	5.45	5.57	0.054
Slc6a1	8.67	8.42	0.462
Pvalb	2.39	1.12	0.017

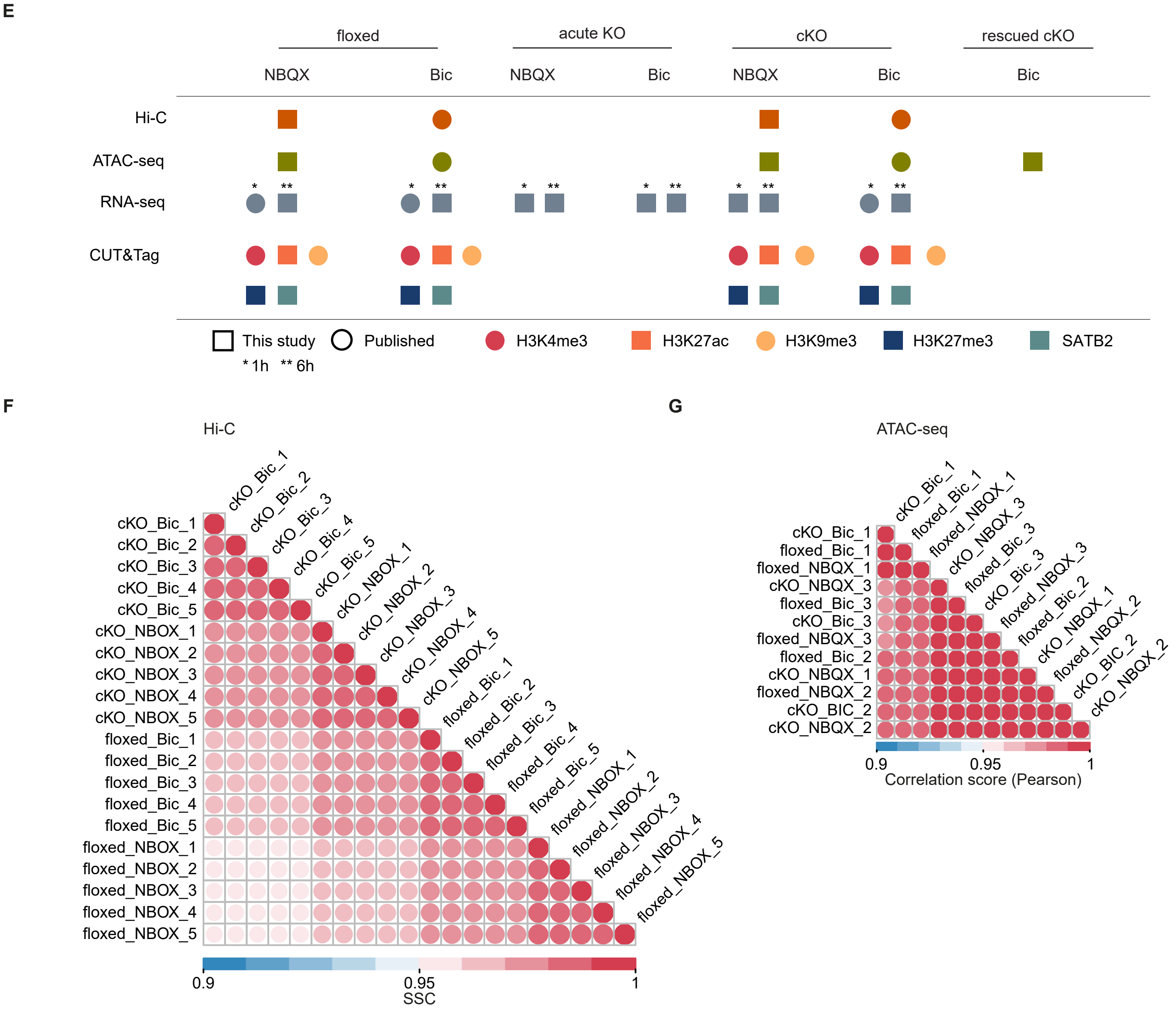

Supplementary Figure 1: Experimental design and analysis pipeline.

A. Schematic illustrating primary cortical neuron differentiation *in vitro*, the timing of genetic manipulations, and stimulation paradigms. Four experimental models were analyzed: control neurons, derived from *Satb2*$^{flx/flx}$ mice (floxed); conditional *Satb2* knockout neurons, derived from *Satb2*$^{flx/flx}$*::Nes-Cre* mice (cKO); acute *Satb2* knockout neurons generated by *in vitro* Cre-mediated recombination (acute KO); and rescued neurons, in which SATB2 expression was restored following conditional deletion by re-expression of SATB2 prior to stimulation (rescued cKO). Neuronal silencing (NBQX) or bicuculline (Bic) stimulation was applied at the indicated time points, and samples were collected for downstream chromatin and transcriptional analyses on day *in vitro* 14 (DIV14). **B.** Representative immunoblots showing SATB2 protein levels in floxed, cKO, acute KO, and rescued cKO cortical neurons. Efficient SATB2 deletion in conditional and acute cKO neurons and restoration of SATB2 expression in rescued neurons are shown. GAPDH was used as a loading control. **C.** Representative immunoblots showing activity-induced expression of NPAS4 and FOS in floxed neurons following Bic stimulation. GAPDH was used as a loading control. **D.** Mean expression levels (log2[TPM+1]) of selected GABA-ergic marker genes in cKO and floxed cultures (n = 13 cKO, n = 12 floxed independent primary culture experiments). Nominal P values are shown; significance threshold after multiple testing correction was $\alpha = 0.0055$. **E.** Schematic summarizing the study design. DIV14 primary cortical cultures were used to generate Hi-C, ATAC-seq, RNA-seq, and CUT&Tag libraries. RNA-seq libraries were collected at 1 h and 6 h following Bic stimulation. Color and shape codes indicate library type and data source (this study or published datasets); asterisks denote post-stimulation time points. **F.** Stratum-adjusted correlation coefficients (SSC) analyzed by HiCRep across replicate Hi-C libraries from this study and Wahl et al. (2024). **G.** Pearson correlation coefficients of read counts between ATAC-seq library replicates from this study and Wahl et al. (2024).

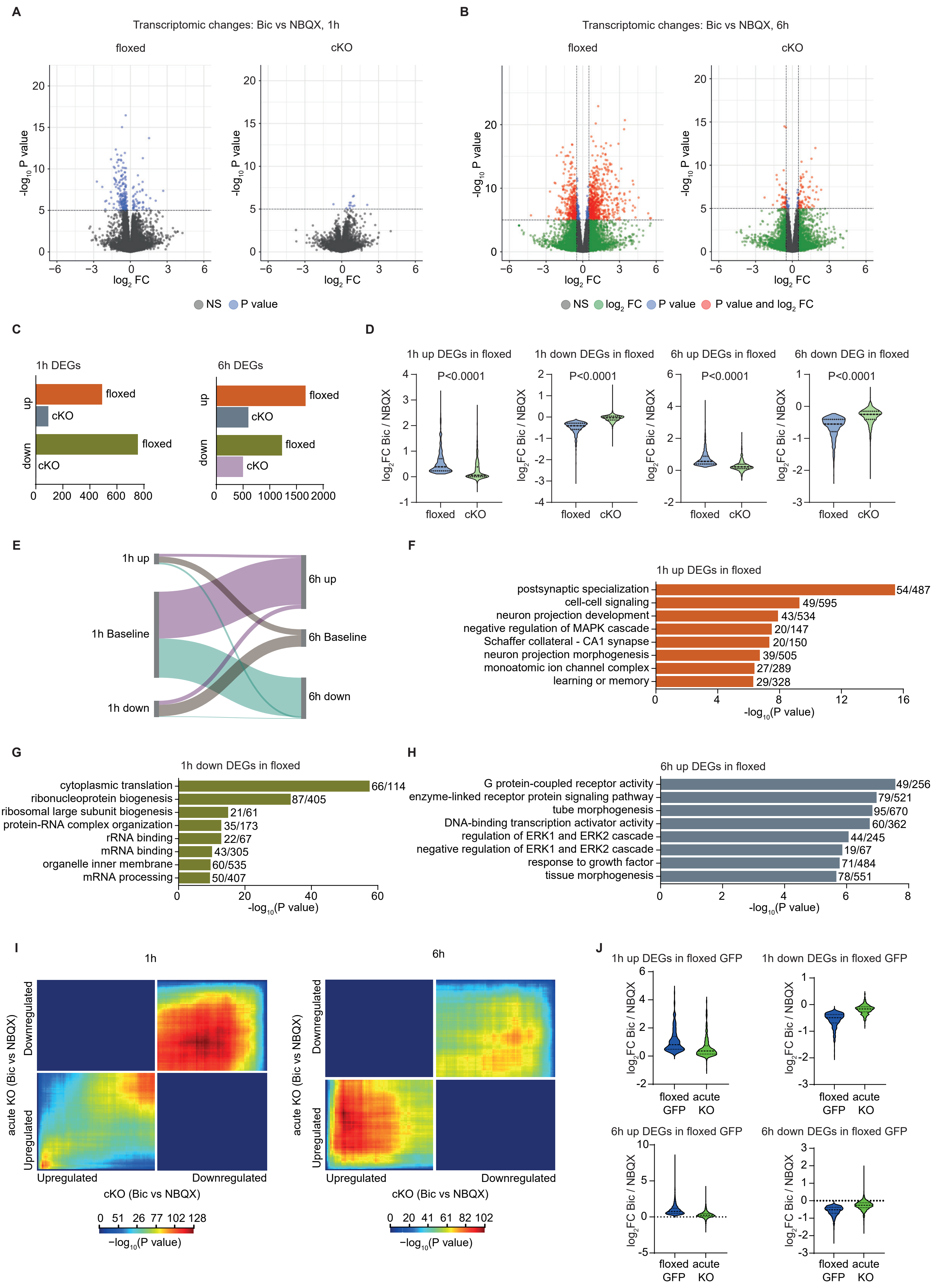
A
Transcriptomic changes: Bic vs NBQX, 1h
floxed
cKO
-log10 P value
log2 FC
NS
P value
B
Transcriptomic changes: Bic vs NBQX, 6h
floxed
cKO
NS
log2 FC
P value
P value and log2 FC
C
1h DEGs
6h DEGs
up
down
floxed
cKO
D
1h up DEGs in floxed
1h down DEGs in floxed
6h up DEGs in floxed
6h down DEG in floxed
P<0.0001
log2FC Bic / NBQX
E
1h up
1h Baseline
1h down
6h up
6h Baseline
6h down
F
1h up DEGs in floxed
postsynaptic specialization 54/487
cell-cell signaling 49/595
neuron projection development 43/534
negative regulation of MAPK cascade 20/147
Schaffer collateral - CA1 synapse 20/150
neuron projection morphogenesis 39/505
monoatomic ion channel complex 27/289
learning or memory 29/328
-log10(P value)
G
1h down DEGs in floxed
cytoplasmic translation 66/114
ribonucleoprotein biogenesis 87/405
ribosomal large subunit biogenesis 21/61
protein-RNA complex organization 35/173
rRNA binding 22/67
mRNA binding 43/305
organelle inner membrane 60/535
mRNA processing 50/407
-log10(P value)
H
6h up DEGs in floxed
G protein-coupled receptor activity 49/256
enzyme-linked receptor protein signaling pathway 79/521
tube morphogenesis 95/670
DNA-binding transcription activator activity 60/362
regulation of ERK1 and ERK2 cascade 44/245
negative regulation of ERK1 and ERK2 cascade 19/67
response to growth factor 71/484
tissue morphogenesis 78/551
-log10(P value)
I
1h
6h
acute KO (Bic vs NBQX)
Downregulated
Upregulated
cKO (Bic vs NBQX)
0 51 26 77 102 128
0 20 41 61 82 102
−log10(P value)
J
1h up DEGs in floxed GFP
1h down DEGs in floxed GFP
6h up DEGs in floxed GFP
6h down DEGs in floxed GFP
log2FC Bic / NBQX
floxed GFP
acute KO

Supplementary Figure 2: Activity-driven transcriptome responses are blunted in *Satb2* cKO cultures

A., B. Volcano plots showing differential gene expression at 1 h **(A)** and 6 h **(B)** following Bic stimulation in primary cortical neurons derived from floxed and cKO cultures. Gene models and RIKEN genes are not shown. **C.** Bar plots showing the number of differentially expressed genes (DEGs), stratified by direction of regulation, in floxed and cKO neurons. **D.** Violin plots showing $\log_2$ fold changes ($\log_2$FC) of up- and downregulated genes identified in floxed neurons at 1 h and 6 h post-stimulation, and the corresponding expression changes of these genes in cKO neurons. Statistical significance was assessed using the Mann–Whitney tests. **E.** Alluvial plot, depicting the temporal dynamics of activity-induced gene expression in floxed neurons at 1 h and 6 h following Bic stimulation. **F., G.** Gene Ontology (GO) enrichment analysis of upregulated **(F)** and downregulated genes **(G)** at 1 h post Bic-stimulation. **H.** GO enrichment analysis of upregulated genes at 6 h post Bic- stimulation. Downregulated genes at 6 h showed no significant enrichment for GO terms. **I.** Rank–rank hypergeometric overlap (RRHO) heatmaps comparing global gene expression signatures ranked by differential expression between Bic- and NBQX-treated neurons at 1 h (left) and 6 h (right) in acute KO versus cKO neurons. Genes were ranked by differential expression P values, effect size and direction. Overlap significance is shown as $-\log_{10}$-transformed hypergeometric P values; the color scale indicates the range of significance. **J.** Violin plots showing expression changes of up- and downregulated genes identified in AAV-GFP transduced floxed neurons at 1 h and 6 h post-stimulation, and the expression changes of these genes in the acute KO model. Statistical significance was assessed using the Mann–Whitney tests.

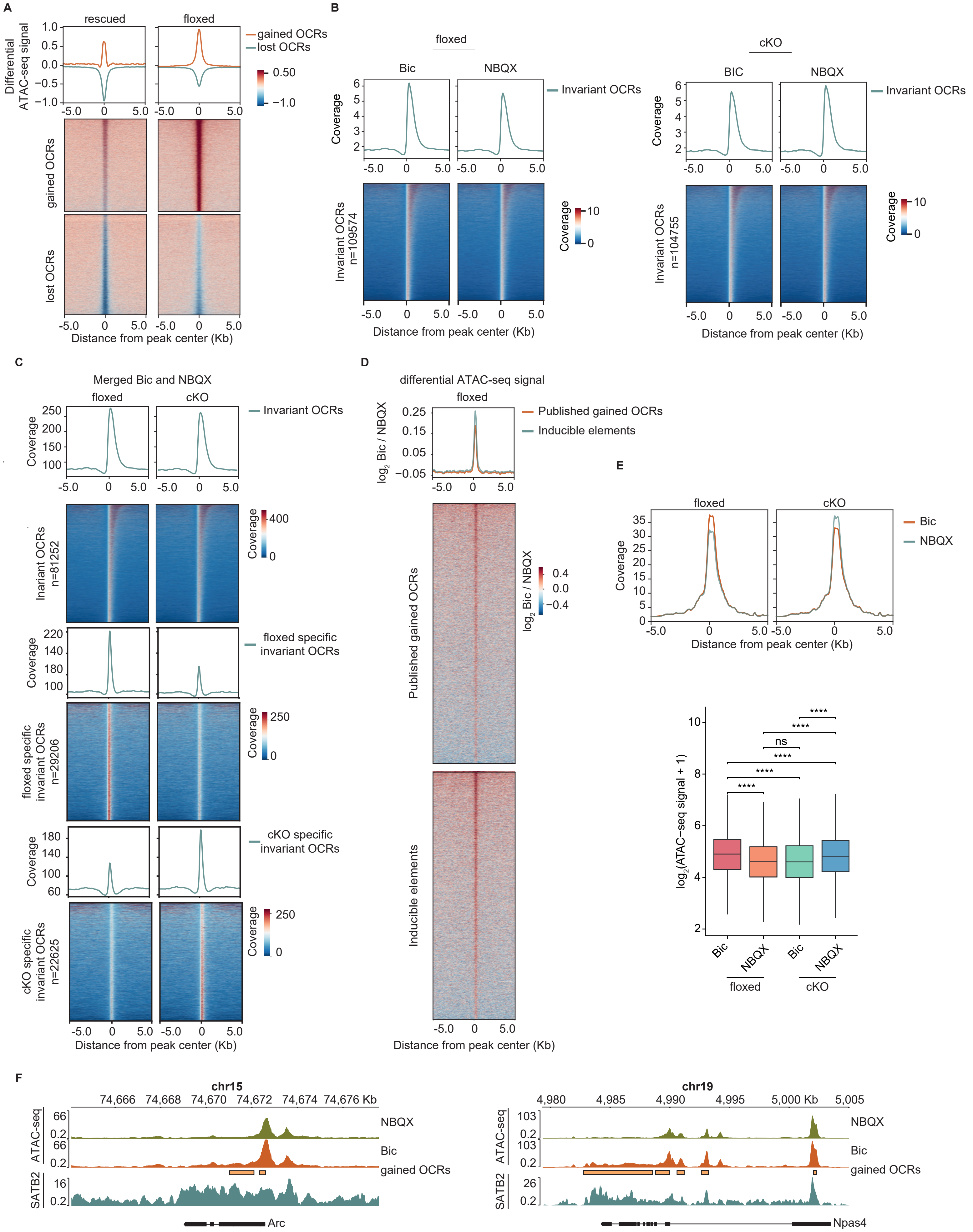

A
rescued
floxed
Differential ATAC-seq signal
gained OCRs
lost OCRs
0.50
−1.0
gained OCRs
lost OCRs
Distance from peak center (Kb)
B
floxed
cKO
Bic
NBQX
BIC
Invariant OCRs
Coverage
Invariant OCRs n=109574
Invariant OCRs n=104755
Distance from peak center (Kb)
C
Merged Bic and NBQX
floxed
cKO
Invariant OCRs
Inariant OCRs n=81252
floxed specific invariant OCRs
floxed specific invariant OCRs n=29206
cKO specific invariant OCRs
cKO specific invariant OCRs n=22625
Distance from peak center (Kb)
D
differential ATAC-seq signal
floxed
Published gained OCRs
Inducible elements
log2 Bic / NBQX
Distance from peak center (Kb)
E
floxed
cKO
Bic
NBQX
Coverage
Distance from peak center (Kb)
log2(ATAC−seq signal + 1)
****
ns
floxed
cKO
F
chr15
74,666 74,668 74,670 74,672 74,674 74,676 Kb
ATAC-seq
NBQX
Bic
gained OCRs
SATB2
Arc
chr19
4,980 4,985 4,990 4,995 5,000 Kb 5,005
Npas4

Supplementary Figure 3: Characterization of invariant and activity-dependent open chromatin regions in cortical neurons

A. Average profiles and heatmaps showing of differential ATAC-seq signals between rescued cKO and floxed cultures (left) and between cKO and floxed cultures (right). The analysis is centered on OCRs, previously defined as gained and lost in the cKO model (Wahl et al., 2024)**. B.** Average profiles and heatmaps showing ATAC-seq signal at invariant OCRs. The plots compare signal in Bic- versus NBQX-treated cultures for both floxed (left) and Satb2 cKO (right) neurons. Data represent merged signal from three replicates (n =3). **C.** Average profiles and heatmaps of merged ATAC-seq signal (Bic and NBQX combined) at different classes of activity-invariant OCRs. Plots are shown for all invariant OCRs (top), floxed-specific invariant OCRs (middle), and *Satb2* cKO-specific invariant OCRs (bottom), highlighting genotype-dependent accessibility as previously reported. (Wahl et al., 2024). Data represent merged signals from three replicates (n = 3). **D.** Average profiles and heatmaps of differential ATAC-seq signal ($\log_2$ Bic/NBQX) in floxed neurons. The analysis is centered on previously published activity-gained OCRs (**top**) and a set of known activity-inducible elements (bottom) (Pollina et al., 2023)**. E.** Analysis of chromatin accessibility at the subset of OCRs that are gained upon stimulation in floxed neurons but lost in cKO neurons. (Top) Average ATAC-seq coverage profiles in floxed (left) and cKO (right) cultures under Bic and NBQX conditions. (Bottom) Quantification of the log-transformed ATAC-seq signal (log2(signal+1) across all four conditions. The data reveal a significantly higher baseline accessibility (NBQX condition) in cKO neurons compared to floxed controls, explaining the apparent "reversal" in activity-dependent response (Kruskal–Wallis test with BH correction for multiple comparisons; ns, not significant, ****P < 0.0001). **F.** Representative genome browser tracks for the activity-induced genes *Arc* (left) and *Npas4* (right). Tracks display ATAC-seq signal in NBQX- and Bic-treated floxed cultures, along with SATB2 CUT&Tag signal. Annotations for activity-gained OCRs (orange) and an invariant SATB2 peak (green) are shown below.

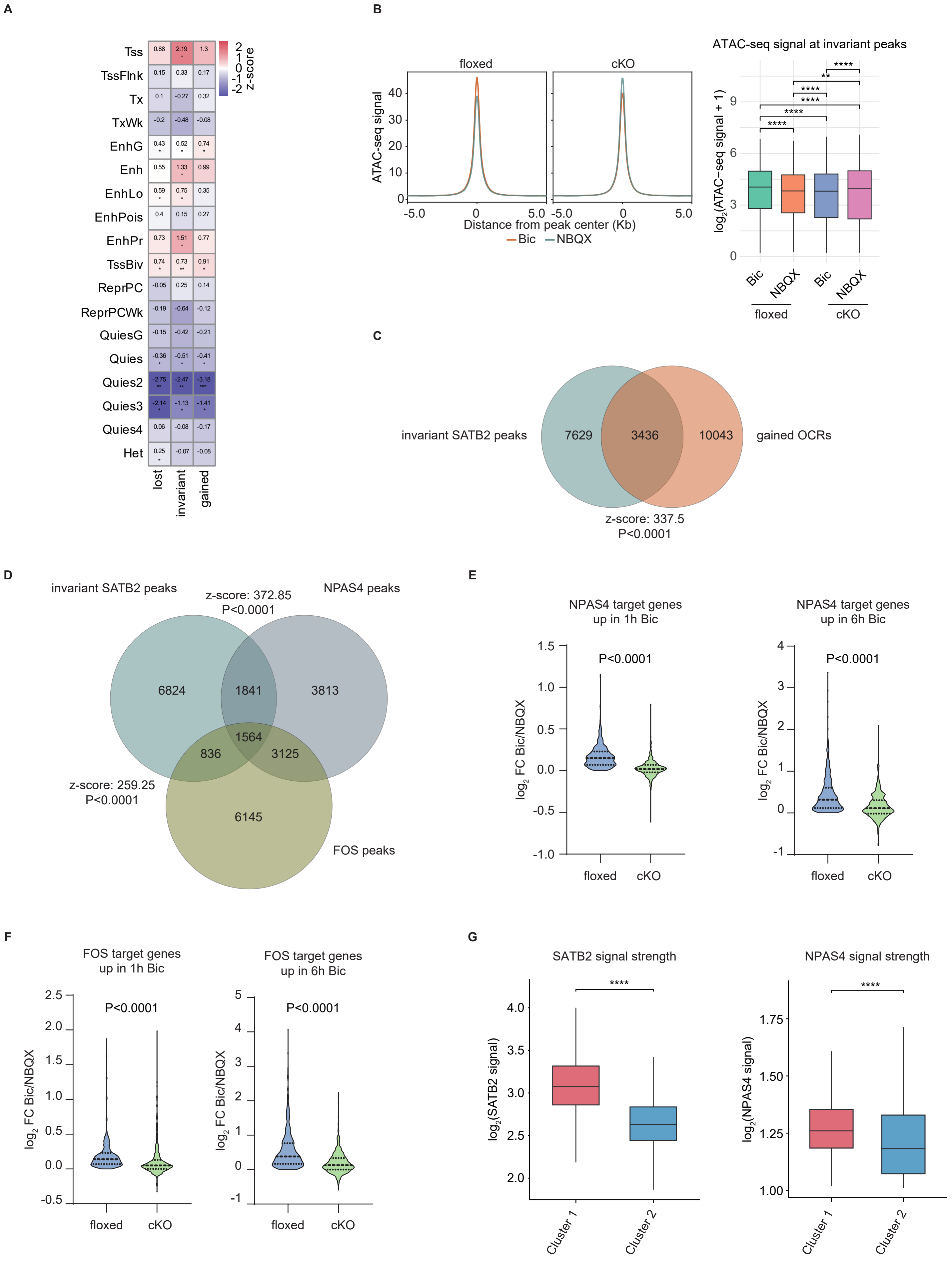
A
Tss
TssFlnk
Tx
TxWk
EnhG
Enh
EnhLo
EnhPois
EnhPr
TssBiv
ReprPC
ReprPCWk
QuiesG
Quies
Quies2
Quies3
Quies4
Het
lost
invariant
gained
z-score
B
floxed
cKO
ATAC-seq signal
Distance from peak center (Kb)
Bic
NBQX
ATAC-seq signal at invariant peaks
log2(ATAC-seq signal + 1)
C
invariant SATB2 peaks
7629
3436
10043
gained OCRs
z-score: 337.5
P<0.0001
D
invariant SATB2 peaks
z-score: 372.85
P<0.0001
NPAS4 peaks
6824
1841
3813
1564
836
3125
6145
z-score: 259.25
P<0.0001
FOS peaks
E
NPAS4 target genes
up in 1h Bic
NPAS4 target genes
up in 6h Bic
P<0.0001
log2 FC Bic/NBQX
floxed
cKO
F
FOS target genes
up in 1h Bic
FOS target genes
up in 6h Bic
P<0.0001
log2 FC Bic/NBQX
floxed
cKO
G
SATB2 signal strength
NPAS4 signal strength
log2(SATB2 signal)
log2(NPAS4 signal)
Cluster 1
Cluster 2

Supplementary Figure 4: Epigenetic features of invariant and activity-dependent SATB2 binding sites in cortical neurons

A. Heatmap showing the enrichment of different SATB2 binding site categories (lost, invariant, gained) across 18 chromatin states from a neonatal forebrain ChromHMM model. The color scale indicates z-scores, and asterisks denote significant enrichments. **B** Analysis of chromatin accessibility at the subset of invariant SATB2 peaks that overlap with activity-gained OCRs. (Left) Average ATAC-seq profiles in floxed and Satb2 cKO cultures. (Right) Box plot quantification of log-transformed ATAC-seq signal (log2(signal+1). The data show reduced chromatin accessibility in Satb2 cKO neurons upon stimulation (Kruskal-Wallis test with BH correction for multiple comparisons; *, $p<0.05$; **, $p<0.01$; ***, $p<0.001$; ****, $p<0.0001$). **C.** Venn diagram showing the significant overlap between invariant SATB2 peaks and activity-gained OCRs (hypergeometric test). **D.** Venn diagram showing the significant overlap between invariant SATB2 peaks, NPAS4 binding sites, and FOS binding sites (hypergeometric test; Pollina et al., 2023). **E.** Violin plots showing that the activity-induced expression of NPAS4 target genes is impaired in Satb2 cKO neurons. Expression change (log2(FC Bic/NBQX) is shown at 1 h (left) and 6 h (right) post-stimulation (paired Wilcoxon test). **F.** Violin plots showing that the activity-induced expression of *FOS* target genes is impaired in *Satb2* cKO neurons, presented as in (E) (paired Wilcoxon test). **G.** Box plots comparing binding strength at bivalent (Cluster 1) versus non-bivalent (Cluster 2) promoters. (Left) SATB2 binding strength from CUT&Tag. (Right) NPAS4 binding strength from CUT&RUN (data from Pollina et al., 2023). Statistical significance was assessed using a Mann-Whitney test.

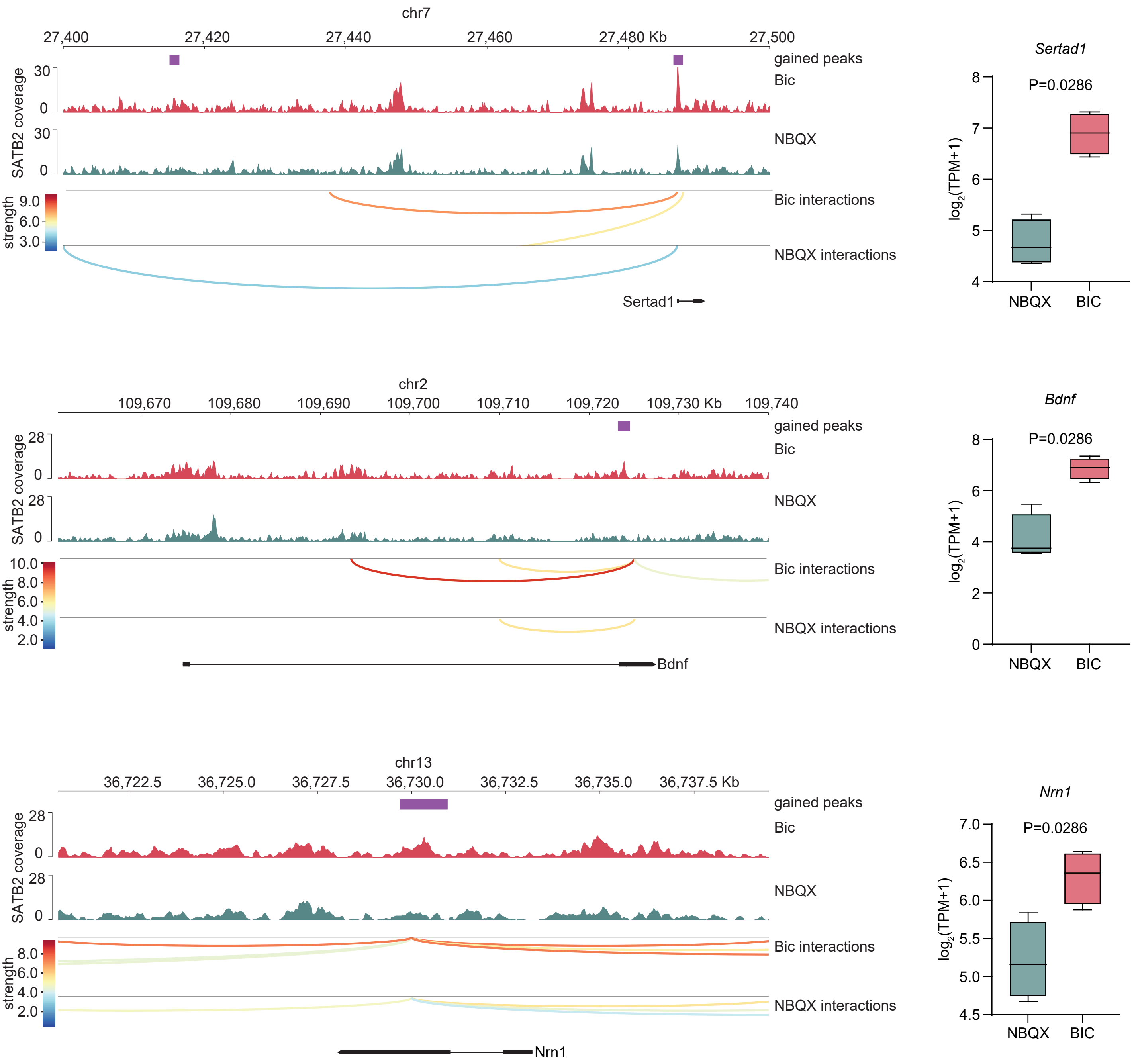

Supplementary Figure 5: Gained SATB2 peaks correlate with enhanced chromatin looping and transcriptional activation at representative delayed primary response loci.

Shown are three representative examples of delayed primary response genes that are upregulated at 6h post-stimulation - *Sertad1* (top), *Bdnf* (middle), and *Nrn1* (bottom) - providing locus-specific visualization for the aggregate data in Figure 4E. For each representative locus, the left panels show SATB2 Cut&Tag coverage tracks in stimulated (Bic, red) versus silenced (NBQX, teal) conditions and Hi-C interaction arcs, which visualize an increase in the strength and extent of local chromatin contacts upon stimulation. A "gained peak" is marked with a purple box. The right panels show gene expression boxplots quantifying $log_2(TPM+1)$ values from RNA-seq. For all three examples, gene expression is significantly increased in the Bic-treated condition compared to NBQX (Mann–Whitney test).

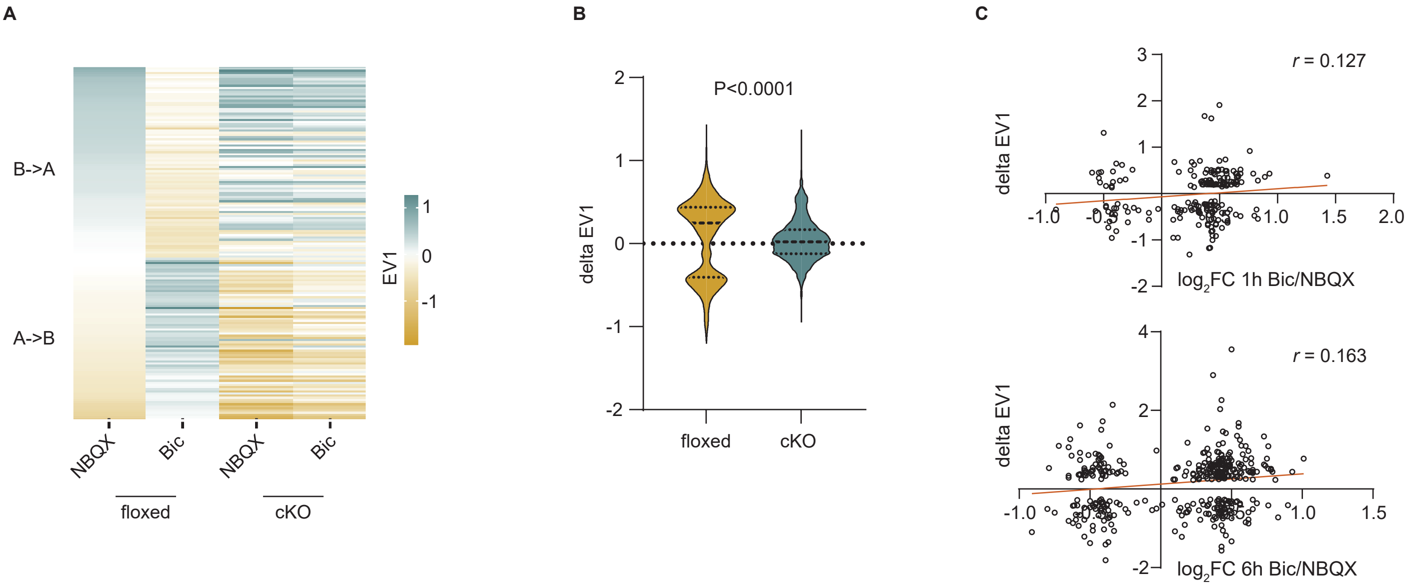

Supplementary Figure 6: A/B compartment changes do not correlate with activity-induced gene expression changes

A. Heatmap showing first eigenvector (EV1) values for genomic regions that undergo compartment switching upon 1h Bic treatment. In floxed neurons, clear B-to-A (gold to teal) and A-to-B (teal to gold) switches are observed. These same regions remain static in *Satb2* cKO cultures, demonstrating that A/B compartment dynamics are dependent on SATB2. **B.** Violin plot quantifying the magnitude of compartment score changes (ΔEV1) following stimulation. The significantly broader distribution of changes in floxed neurons confirms a dynamic compartment landscape that is lost upon *Satb2* deletion (paired non-parametric Wilcoxon test). **C** Scatter plots testing for a correlation between compartment score changes (ΔEV1) and gene expression changes ($\log_2$ fold change) in floxed neurons. The analysis at both 1h (top) and 6h (bottom) post-stimulation reveals only weak correlations, indicating that activity-dependent A/B compartment switching is not the primary driver of the observed transcriptional changes.

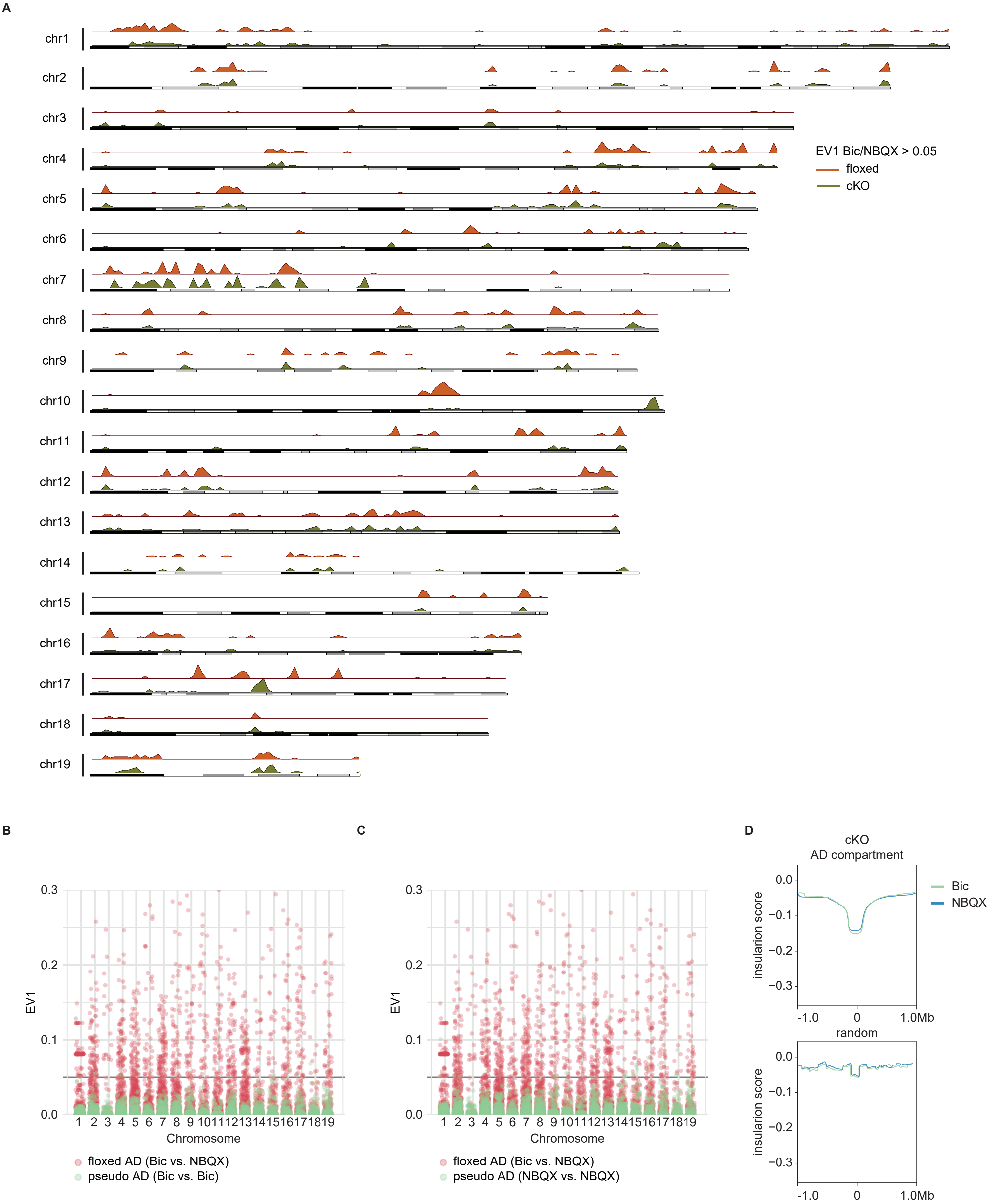
A
chr1
chr2
chr3
chr4
chr5
chr6
chr7
chr8
chr9
chr10
chr11
chr12
chr13
chr14
chr15
chr16
chr17
chr18
chr19
EV1 Bic/NBQX > 0.05
floxed
cKO
B
EV1
0.3
0.2
0.1
0.0
1 2 3 4 5 6 7 8 9 10 11 12 13 14 15 16 17 18 19
Chromosome
floxed AD (Bic vs. NBQX)
pseudo AD (Bic vs. Bic)
C
EV1
0.3
0.2
0.1
0.0
1 2 3 4 5 6 7 8 9 10 11 12 13 14 15 16 17 18 19
Chromosome
floxed AD (Bic vs. NBQX)
pseudo AD (NBQX vs. NBQX)
D
cKO
AD compartment
insulation score
0.0
−0.1
−0.2
−0.3
-1.0
0
1.0Mb
Bic
NBQX
random
insulation score
0.0
−0.1
−0.2
−0.3
-1.0
0
1.0Mb

Supplementary Figure 7: The AD compartment is a robust, 3D genome architectural feature requiring SATB2. A. Genome-wide view across all autosomes showing the Activity-dependent (AD) compartment signal in floxed (orange) and *Satb2* cKO (dark olive green) cultures. The signal is defined by the first eigenvector (EV1 > 0.05) from differential Hi-C matrices (Bic vs. NBQX). These tracks illustrate the widespread nature of the AD compartment and its impaired formation in the absence of SATB2. **B, C.** Technical validation of the AD compartment calling method. Biological replicates from each condition were divided into two separate, depth-matched pools (n=3 per pool) to perform a "within-condition" comparison. The plots show the resulting negligible AD compartment scores from comparing **(B)** two pools of Bic replicates and **(C)** two pools of NBQX replicates. This confirms the AD signal is a robust biological finding, not a technical artifact. **D**. Aggregate insulation score plots centered on the boundaries of AD compartments defined within the cKO genotype (top). These boundaries show a modest baseline insulation dip (NBQX) but exhibit a complete absence of activity-dependent strengthening upon stimulation (Bic). A control at random genomic locations (bottom) is shown below for comparison.

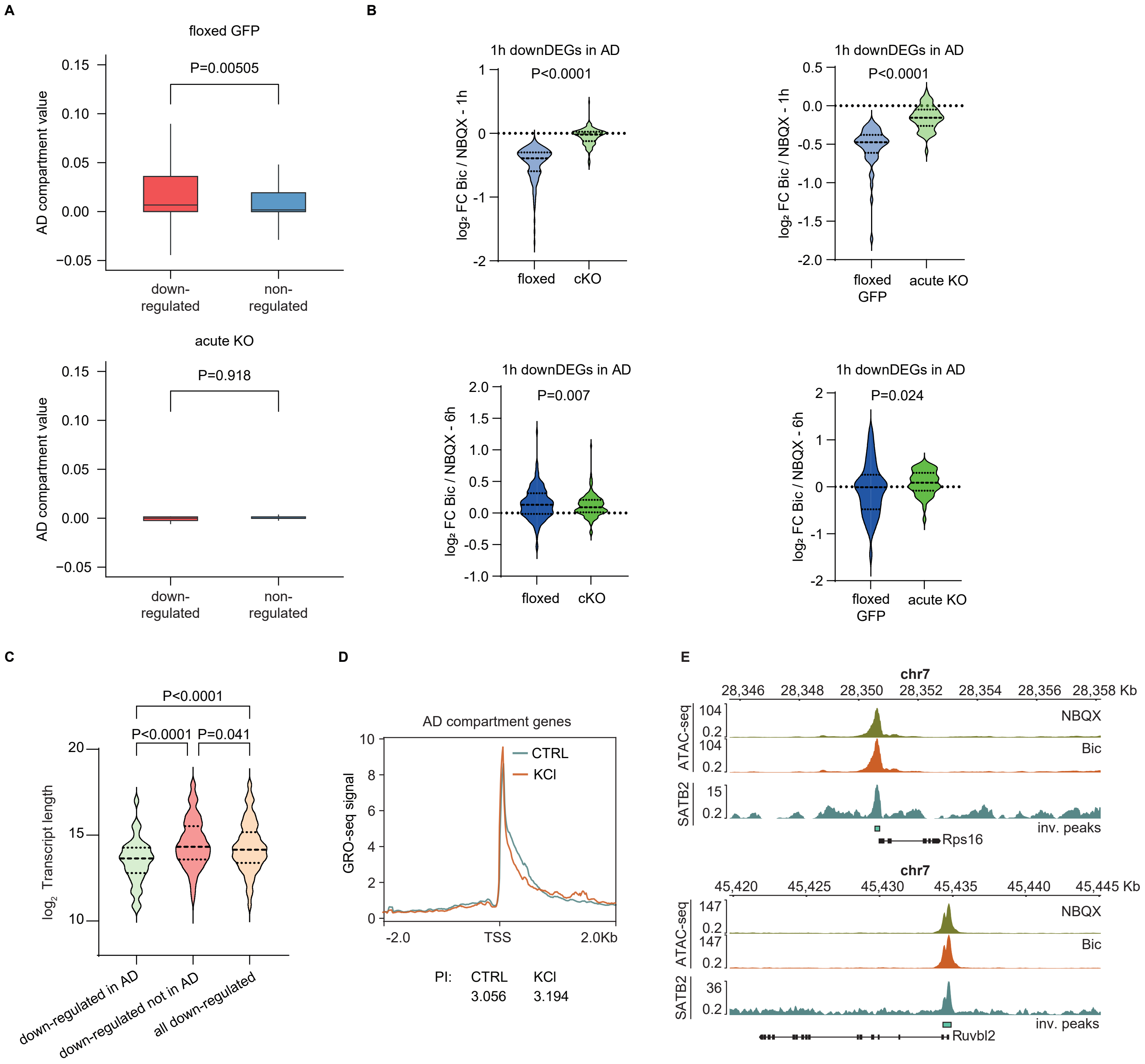

Supplementary Figure 8: Neuronal activity-triggered downregulation of genes harbored in the AD compartment is also impaired in acute *Satb2* cKO deletion model.

A. Box plots comparing the AD compartment value of downregulated versus non-regulated genes. In control (floxed GFP-transduced) neurons, downregulated genes are associated with higher AD compartment values (top). This association is lost in the acute Satb2 KO model (bottom) (Permutation test, n=1,000). **B.** Violin plots comparing expression changes (log2(FC Bic/NBQX) of genes downregulated at 1 h in the AD compartment. The analysis compares the chronic versus acute effects of Satb2 loss. Left column: Chronic Satb2 cKO model. Expression changes at 1 h (top) and 6 h (bottom) post-stimulation. Right column: Acute Satb2 KO model. Expression changes at 1 h (top) and 6 h (bottom) post-stimulation. Note the impaired downregulation at 1 h in both models and the transient nature of the effect (Mann-Whitney). **C.** Violin plots comparing log-transformed transcript lengths in the acute *Satb2* KO model. The analysis compares downregulated genes inside the AD compartment, outside the AD compartment, and the set of all downregulated genes at 1 h post-stimulation (Kruskal-Wallis test). **D**. Average GRO-seq profiles at genes mapped to invariant SATB2 peaks in the AD compartment. Profiles from mouse primary cortical neurons are shown at baseline (CTRL) and 1 h post-KCl stimulation, centered on the transcription start site (TSS ±2 kb). The pausing index (PI) is calculated below as the ratio of promoter-proximal to gene body GRO-seq signal (data from Gorbovytska et al., 2022). **E.** Representative genome browser tracks for the genes *Rps16* and *Ruvbl2*, which are mapped to invariant SATB2 peaks in the AD compartment. Tracks show unchanged ATAC-seq signal between NBQX and Bic conditions, SATB2 CUT&Tag signal, and the location of invariant SATB2 peaks.

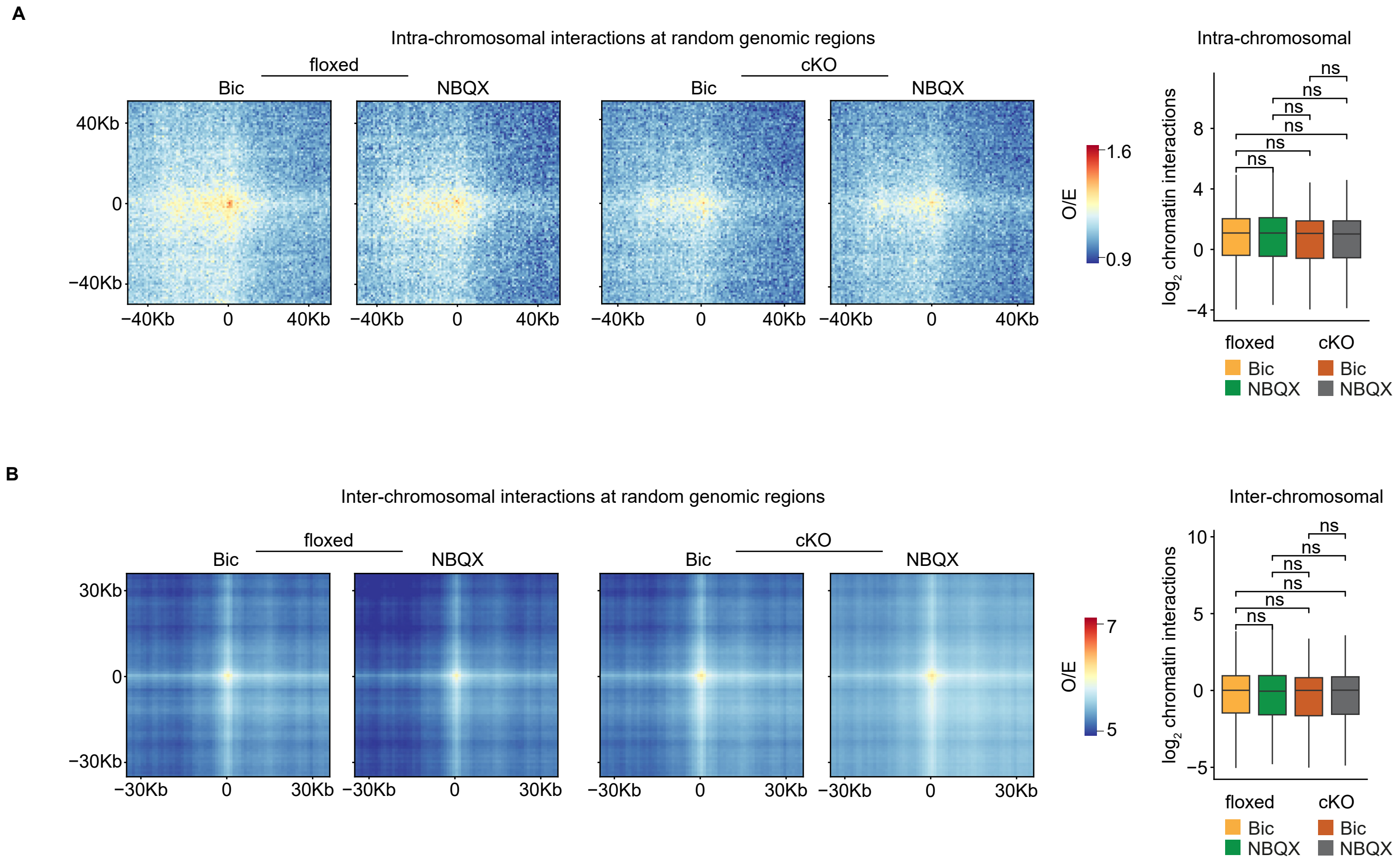

Supplementary Figure 9: Control aggregate analyses at random genomic regions show no activity-dependent changes

(**A**) Aggregate pile-up maps showing intra-chromosomal contact enrichment (O/E scores) centred on random genomic regions matched in size and number to invariant SATB2 peaks in AD. The boxplot (right) quantifies log_2-transformed interaction strength and shows no significant changes across genotypes or treatments. **(B)** Aggregate pile-up maps of inter-chromosomal contacts (O/E scores) centred on random genomic regions matched in size and number to invariant SATB2 peaks in AD. The boxplot (right) likewise shows no significant changes in interaction strength.

Statistical analysis for all panels: Kruskal-Wallis test with Benjamini-Hochberg correction; ns, not significant.
